# The Genetic Landscape of Colorectal Cancer: From Molecular Alterations to Therapeutic Decision Pathways

**DOI:** 10.3390/cancers18152526

**Published:** 2026-08-06

**Authors:** Cristina Maria Macrea, Tiberia Ilias, Alexandra Costea, Paula Trif, Viorela-Romina Murvai, Ovidiu C. Fratila

**Affiliations:** 1 Doctoral School of Biological and Biomedical Sciences, University of Oradea, 410087 Oradea, Romania; 2 Department of Medical Disciplines, Faculty of Medicine and Pharmacy, University of Oradea, 410087 Oradea, Romania; 3 Department of Preclinical Disciplines, Faculty of Medicine and Pharmacy, University of Oradea, 410087 Oradea, Romania; 4 Department of Obstetrics and Gynecology, Calla—Infertility Diagnostic and Treatment Center, Constantin A. Rosetti Street, 410103 Oradea, Romania

**Keywords:** colorectal cancer, precision oncology, molecular biomarkers, MSI, KRAS, BRAF, HER2, NTRK, ctDNA, tumor mutational burden, transcriptomics, artificial intelligence, personalized medicine

## Abstract

Colorectal cancer (CRC) remains one of the leading causes of cancer-related morbidity and mortality worldwide despite substantial advances in screening, surgical techniques, systemic therapies, and multidisciplinary care. The increasing implementation of precision oncology has fundamentally transformed CRC management by enabling molecularly guided therapeutic strategies based on tumor-specific genetic alterations. In recent years, the molecular landscape of CRC has expanded considerably beyond traditional histopathological classification, incorporating a growing number of clinically actionable biomarkers with prognostic, predictive, and therapeutic significance. This review provides a comprehensive and up-to-date overview of the genetic landscape of CRC, focusing on established biomarkers currently integrated into clinical practice, including microsatellite instability/mismatch repair deficiency (MSI/dMMR), *KRAS*, *NRAS*, *BRAF*, HER2, and *NTRK* alterations. In addition, emerging biomarkers such as tumor mutational burden (TMB), *POLE/POLD1* mutations, circulating tumor DNA (ctDNA), DNA damage repair (DDR) alterations, transcriptomic signatures, and artificial intelligence-based molecular prediction models are critically discussed. Particular emphasis is placed on their biological significance, diagnostic methodologies, prognostic and predictive value, and potential role in treatment selection. A structured, database-informed narrative review identified 140 relevant publications, primarily published between January 2020 and June 2026, supplemented by earlier seminal studies and major clinical guidelines. Based on the available evidence, we propose a Clinical Actionability Framework for CRC, categorizing biomarkers into three hierarchical tiers according to their level of clinical validation and therapeutic relevance: established standard-of-care biomarkers, emerging clinical biomarkers, and future precision oncology biomarkers. Collectively, current evidence supports a progressive transition from single-gene testing toward integrated multi-omics precision medicine. Advances in comprehensive genomic profiling, liquid biopsy technologies, transcriptomics, radiogenomics, and artificial intelligence are expected to further refine patient stratification, optimize therapeutic decision-making, and facilitate the development of adaptive precision oncology models. Understanding the evolving genetic landscape of CRC is therefore essential for maximizing treatment efficacy and improving patient outcomes in the era of personalized cancer care.

## Simple Summary

Colorectal cancer is no longer considered a single disease but rather a collection of biologically distinct tumor subtypes characterized by specific genetic alterations. These molecular differences have transformed patient management by enabling personalized treatment strategies based on individual tumor profiles. This review summarizes the most clinically relevant genetic biomarkers currently used in colorectal cancer, including microsatellite instability, *RAS* and *BRAF* mutations, HER2 amplification, and *NTRK* fusions, while also discussing emerging biomarkers such as circulating tumor DNA, tumor mutational burden, transcriptomic signatures, microbiome-derived markers, epigenetic alterations, and artificial intelligence-based prediction models. We propose a practical framework that categorizes biomarkers according to their current clinical applicability and future translational potential. Understanding how these biomarkers influence diagnosis, prognosis, treatment selection, and disease monitoring may help clinicians and researchers navigate the rapidly evolving landscape of precision oncology and support the development of more individualized approaches for patients with colorectal cancer.

## 1. Introduction

Colorectal cancer (CRC) remains one of the most frequently diagnosed malignancies and a leading cause of cancer-related mortality worldwide. Despite significant advances in screening programs, surgical techniques, systemic therapies, and multidisciplinary management, CRC continues to impose a substantial global health burden [1,2,3]. Recent global estimates confirm that CRC continues to impose a substantial burden of incidence and mortality, reinforcing the need for improved prevention, early detection, risk stratification, and personalized therapeutic strategies [4].

The development and progression of CRC are driven by a complex accumulation of genetic and epigenetic alterations that affect multiple cellular pathways involved in proliferation, apoptosis, DNA repair, angiogenesis, and immune surveillance. Traditionally, colorectal carcinogenesis has been described through three major molecular pathways: chromosomal instability (CIN), microsatellite instability (MSI), and the CpG island methylator phenotype (CIMP). Among these, the CIN pathway accounts for the majority of sporadic CRC cases and is characterized by sequential alterations involving genes such as APC, TP53, and KRAS. In contrast, MSI results from defects in the DNA mismatch repair (MMR) system and is present in approximately 10–15% of CRCs, while epigenetic dysregulation contributes substantially to tumor heterogeneity and disease progression [1,2,3].

The integration of molecular biomarkers into routine clinical practice has transformed CRC management, enabling personalized therapeutic strategies and improving patient stratification. Biomarker-guided approaches now influence treatment selection, prognosis assessment, and therapeutic monitoring across multiple stages of disease.

Among currently established biomarkers, MSI-H/dMMR status has emerged as one of the most clinically relevant predictors of response to immunotherapy. The remarkable efficacy of immune checkpoint inhibitors in MSI-H metastatic CRC has fundamentally changed treatment paradigms and demonstrated the value of genomic profiling in therapeutic selection. However, MSI-H tumors represent only a minority of CRC cases, whereas the majority of patients harbor microsatellite-stable (MSS) disease, which remains relatively resistant to immunotherapeutic approaches. This limitation has stimulated extensive research into additional genomic, transcriptomic, and microenvironmental biomarkers capable of improving patient stratification and predicting treatment response [3].

Simultaneously, technological advances in next-generation sequencing (NGS), liquid biopsy, circulating tumor DNA (ctDNA) analysis, artificial intelligence-assisted pathology, and multi-omics profiling have expanded the molecular landscape of CRC beyond traditional single-gene testing. Comprehensive genomic profiling now enables the simultaneous assessment of multiple actionable alterations, facilitating more precise molecular classification and supporting the transition toward individualized treatment algorithms. Emerging evidence suggests that future clinical decision-making will increasingly rely on integrated molecular signatures rather than isolated biomarkers alone.

According to the Global Cancer Observatory, GLOBOCAN 2024 estimated 1,206,011 new cases of colon cancer and 778,594 new cases of rectal cancer worldwide. Taken together, these estimates correspond to approximately 1.98 million newly diagnosed colorectal cancers in a single year. Colon and rectal cancers were estimated to cause 556,774 and 339,370 deaths, respectively, resulting in a combined mortality burden of approximately 896,000 deaths. The corresponding five-year prevalence was approximately 3.41 million for colon cancer and 2.33 million for rectal cancer, equivalent to more than 5.74 million individuals living within five years of a CRC diagnosis worldwide. These data demonstrate the substantial global burden of CRC and reinforce the importance of improving prevention, molecular stratification, and access to effective biomarker-guided therapies. According to GLOBOCAN 2024 estimates, colon and rectal cancers together accounted for approximately 1.98 million new cases and 896,000 deaths worldwide [4].

Given the rapidly evolving therapeutic landscape and the growing complexity of molecular diagnostics, a comprehensive synthesis of currently established and emerging genetic biomarkers is warranted. Therefore, this review aims to provide an updated overview of the genetic landscape of CRC, focusing on clinically actionable molecular alterations, their biological significance, diagnostic methodologies, prognostic and predictive value, and their integration into contemporary precision oncology frameworks. Furthermore, we discuss emerging technologies and future directions that are expected to shape the next generation of personalized CRC management [3,5].

## 2. Materials and Methods

### 2.1. Study Design

This article was conducted as a structured narrative review intended to provide a clinically oriented synthesis of established and emerging molecular alterations in CRC. It was not designed as a systematic review or meta-analysis. Accordingly, no review protocol was prospectively registered, no formal duplicate screening process was performed, and no standardized risk-of-bias instrument was applied. The preparation of the review was informed by the principles of transparent narrative-review reporting, including a clear justification of the review question, description of the literature-identification approach, appropriate referencing of key statements, and balanced presentation of the available evidence.

### 2.2. Literature Identification and Source Selection

A database-informed literature search was conducted in PubMed/MEDLINE, Scopus, Web of Science Core Collection, and Embase, supplemented by targeted searches in Google Scholar and manual examination of the reference lists of relevant publications. The search primarily focused on literature published between January 2020 and June 2026. Seminal earlier publications and major clinical guidelines were also included when required to describe established molecular pathways, hereditary syndromes, classification systems, or practice-changing evidence.

Publications were selected purposively according to their relevance to the biological, diagnostic, prognostic, predictive, or therapeutic implications of molecular alterations in CRC. Priority was given to clinical guidelines, randomized and prospective clinical studies, high-quality systematic reviews and meta-analyses, clinically informative real-world studies, and major translational investigations. The search was intended to identify representative and clinically influential evidence rather than to generate an exhaustive or quantitatively reproducible systematic-review dataset.

### 2.3. Eligibility Criteria

#### 2.3.1. Inclusion Criteria

Studies were considered eligible if they met one or more of the following criteria:

• Published in peer-reviewed journals.
• Written in English.
• Published between 2020 and 2026.
• Focused on CRC molecular biology, genetics, or precision oncology.

Investigated clinically relevant biomarkers, including MSI/dMMR, *KRAS*, *NRAS*, *BRAF*, Human epidermal growth factor receptor 2 (HER2), *NTRK*, TMB, ctDNA, DDR alterations, transcriptomic signatures, or AI-derived biomarkers.

Reported diagnostic, prognostic, predictive, or therapeutic implications of molecular alterations.

Included clinical trials, observational studies, real-world evidence studies, systematic reviews, meta-analyses, translational studies, or molecular profiling investigations.

#### 2.3.2. Exclusion Criteria

Studies were excluded if they met any of the following conditions:

• Non-English publications.
• Conference abstracts without full-text availability.
• Editorials, letters, expert opinions, or commentaries lacking original scientific content.
• Animal-only studies without translational relevance to human CRC.
• Studies focused exclusively on non-colorectal malignancies.
• Publications with insufficient methodological details or unavailable full texts.
• Duplicate publications or overlapping datasets.

### 2.4. Study Selection Process

The initial database search yielded 302 potentially relevant articles. Articles were assessed for thematic relevance using prespecified inclusion considerations consistent with the objectives of this structured narrative review.

Studies were prioritized based on:

• Clinical applicability of investigated biomarkers.
• Relevance to precision oncology and targeted therapies.
• Quality of study design.
• Therapeutic implications.
• Diagnostic methodology.
• Recency of publication.

Following full-text evaluation, 140 articles were selected for final qualitative synthesis. The final dataset included clinical trials, systematic reviews, meta-analyses, real-world evidence studies, translational investigations, and molecular profiling studies addressing the contemporary genetic landscape of CRC.

### 2.5. Data Extraction and Synthesis

For each included study, the following information was extracted:

• Author and publication year.
• Study design.
• Sample size and patient population.
• Molecular biomarkers investigated.
• Detection methodologies.
• Prognostic and predictive findings.
• Therapeutic implications.
• Emerging translational applications.

Given the heterogeneity of study designs, biomarker platforms, and reported outcomes, a qualitative synthesis approach was adopted. Evidence was organized according to biomarker categories and subsequently integrated into a practical CRC-oriented clinical implementation framework informed by established precision-oncology classification systems, particularly ESCAT and OncoKB [6].

### 2.6. Measures to Reduce Selection and Interpretation Bias

Several measures were applied to reduce selection and interpretation bias. First, literature identification was conducted across multiple databases, including PubMed/MEDLINE, Scopus, Web of Science Core Collection, and Embase, supplemented by targeted searches in Google Scholar and manual screening of the reference lists of major guidelines and pivotal publications. This approach was intended to reduce dependence on a single database or indexing system.

Second, publications were considered according to predefined thematic eligibility criteria covering hereditary susceptibility, molecular carcinogenesis, analytically validated biomarkers, therapeutic actionability, molecular residual disease, treatment resistance, and emerging multi-omics technologies. Priority was given to international clinical guidelines, randomized and prospective clinical trials, major translational cohorts, and high-quality meta-analyses. Narrative reviews were used mainly to provide broad biological context rather than to support specific therapeutic efficacy claims when original evidence was available.

Third, the synthesis deliberately incorporated negative, inconclusive, and conflicting evidence. For example, the revised discussion of TMB includes studies showing that isolated TMB-high status in microsatellite-stable CRC does not consistently predict benefit from immune-checkpoint inhibition. This approach was intended to reduce emphasis on positive or confirmatory findings alone.

Following the second-round review, the manuscript underwent a comprehensive claim-to-citation audit. Each substantive statement was reassessed against the scope and findings of the cited publication, and references that were indirect, non-CRC-specific, or unrelated to the corresponding claim were removed or replaced. References concerning other tumor types or only indirectly related biomarker concepts were removed or replaced with CRC-specific primary evidence. Nevertheless, because the review did not include formal duplicate independent screening, protocol registration, or a standardized risk-of-bias assessment, residual selection and interpretation bias cannot be excluded.

### 2.7. CRC-Oriented Clinical Implementation Framework

To facilitate navigation of the heterogeneous biomarker landscape, this review uses a simplified CRC-oriented clinical implementation framework. The framework is informed by established precision-oncology systems, including the ESMO Scale for Clinical Actionability of Molecular Targets and OncoKB, but it is not intended to replace these alteration-specific evidence classifications [6,7,8].

Whereas ESCAT and OncoKB primarily evaluate the evidence supporting specific alteration–tumor–therapy relationships, the present framework organizes broader biomarker domains according to their current function in CRC care. These functions include hereditary-risk assessment, diagnosis, prognosis, treatment selection, treatment exclusion, minimal-residual-disease assessment, resistance monitoring, and future biomarker development. The categories should therefore be interpreted as a practical narrative organization of the review rather than as a newly validated evidence-grading system.

**Figure 1:**
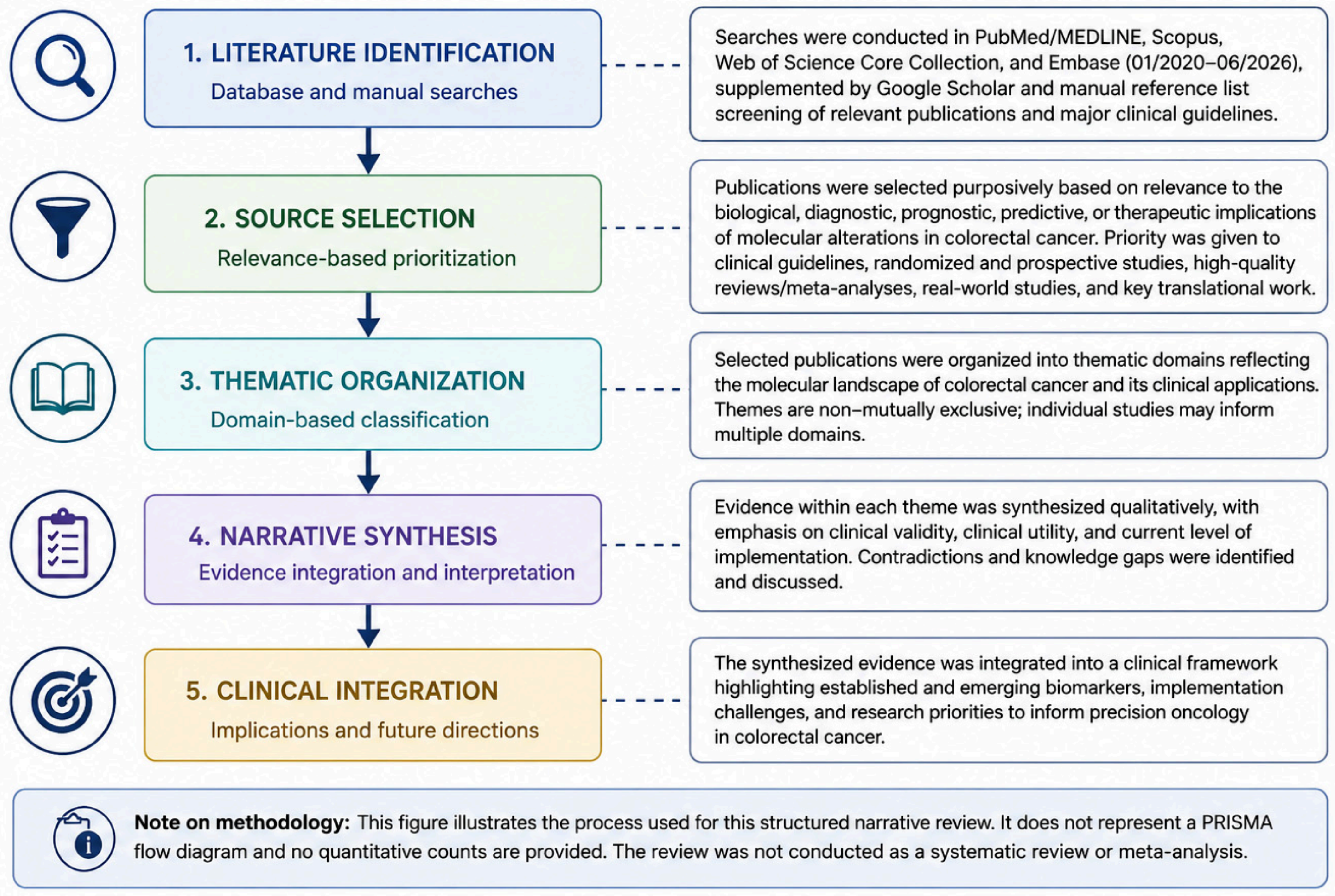
Literature identification and thematic synthesis process used for the structured narrative review.

To provide a transparent overview of the evidence selection process, the study identification, screening, eligibility assessment, and inclusion stages are presented in Figure 1.

## 3. Molecular Pathways of Colorectal Carcinogenesis

Colorectal carcinogenesis is a multistep evolutionary process driven by the progressive accumulation of genetic and epigenetic alterations. Rather than representing a single disease entity, CRC encompasses a heterogeneous group of tumors arising through distinct molecular pathways that differ in their biological behavior, prognostic significance, and therapeutic vulnerabilities. Understanding these pathways is fundamental for the implementation of precision oncology and biomarker-guided treatment strategies [1,3].

Traditionally, three major molecular mechanisms have been recognized in CRC development: chromosomal instability (CIN), microsatellite instability (MSI), and the CpG island methylator phenotype (CIMP). These pathways are not entirely independent and may partially overlap, contributing to the substantial molecular heterogeneity observed among CRC patients.

### 3.1. Chromosomal Instability Pathway

Chromosomal instability represents the predominant molecular pathway in CRC, accounting for approximately 70–85% of sporadic CRC cases. This pathway is characterized by large-scale chromosomal abnormalities, including gains and losses of chromosomal segments, aneuploidy, loss of heterozygosity, and structural rearrangements. These alterations result in the activation of oncogenes and inactivation of tumor suppressor genes, ultimately promoting malignant transformation and tumor progression [1].

The classical adenoma–carcinoma sequence described by Fearon and Vogelstein remains the best-known model of CIN-driven tumorigenesis. Early in this process, inactivating mutations in the *APC* gene lead to dysregulation of the Wnt/β-catenin signaling pathway and uncontrolled cellular proliferation. Subsequent activating mutations in *KRAS* further enhance tumor growth and survival, while later alterations involving *TP53*, *SMAD4*, and other tumor suppressor genes facilitate invasion and metastatic dissemination [3,7].

### 3.2. Microsatellite Instability Pathway

Microsatellite instability results from defects in the DNA mismatch repair (MMR) system, leading to the accumulation of insertion and deletion errors within repetitive DNA sequences known as microsatellites [9]. The MMR machinery is primarily composed of the proteins MLH1, MSH2, MSH6, and PMS2, which are responsible for correcting DNA replication errors and maintaining genomic integrity [10,11,12].

Deficient mismatch repair (dMMR) leads to the hypermutated phenotype characteristic of MSI-high (MSI-H) tumors. Approximately 10–15% of all CRCs exhibit MSI-H status, whereas only about 4–5% of metastatic CRC cases belong to this subgroup. MSI-H tumors may arise either through germline mutations associated with Lynch syndrome or through sporadic epigenetic silencing of the *MLH1* promoter [9]. The hypermutated phenotype of MSI-H tumors contributes to increased immunogenicity and underlies their clinical responsiveness to immune checkpoint inhibitors. This immunogenic microenvironment explains the remarkable sensitivity of MSI-H CRC to immune checkpoint inhibitors targeting the PD-1/PD-L1 axis, which have fundamentally changed treatment paradigms in advanced disease [9,12].

### 3.3. CpG Island Methylator Phenotype

The CpG island methylator phenotype (CIMP) is characterized by widespread promoter hypermethylation resulting in transcriptional silencing of critical regulatory genes. Unlike CIN and MSI, which primarily involve genetic alterations, CIMP reflects epigenetic dysregulation and represents an important mechanism of colorectal tumorigenesis [3,13,14].

CIMP-positive tumors frequently exhibit proximal colon localization, poor differentiation, mucinous histology, and a higher prevalence among older female patients. Notably, CIMP is strongly associated with *BRAF *V600E mutations and often coexists with sporadic MSI-H tumors through methylation-induced silencing of *MLH1*. This molecular association creates a biologically distinct CRC subgroup with unique prognostic and therapeutic implications [13].

Increasing evidence suggests that epigenetic alterations contribute not only to tumor initiation but also to treatment resistance, immune evasion, and metastatic progression. Consequently, epigenetic biomarkers and methylation-based molecular classifications are becoming important areas of translational CRC research [13,14].

### 3.4. Molecular Heterogeneity and Pathway Interactions

Although CRC molecular pathways are commonly described as separate entities, substantial overlap exists between them. For example, MSI-H tumors frequently harbor *BRAF *V600E mutations, while MSS tumors are more commonly associated with *KRAS* alterations. Emerging studies have also identified additional molecular features, including tumor mutational burden (TMB), *POLE/POLD1* mutations, DNA damage repair defects, and transcriptomic subtypes that further refine molecular classification.

Recent advances in next-generation sequencing have revealed that CRC should be viewed as a dynamic evolutionary disease rather than a collection of static genetic alterations. Tumor evolution under selective pressures imposed by therapy can lead to clonal expansion, emergence of resistance mechanisms, and changes in molecular profiles over time. Therefore, contemporary precision oncology increasingly relies on comprehensive genomic profiling and longitudinal molecular monitoring to guide therapeutic decision-making [3,8].

Collectively, these molecular pathways form the biological framework upon which modern biomarker-driven management of CRC is built. The following sections discuss the major actionable genetic alterations currently used in clinical practice and their implications for targeted therapy, immunotherapy, and personalized patient care (Table 1) [3,5].

**Table 1:** Clinical Actionability and Precision Oncology Relevance of Established and Emerging Biomarkers in CRC.

Biomarker	Approximate Prevalence	Recommended Testing Method	Clinical Utility	Therapeutic Implications	Current Implementation Status	Ref
Tier 1—Established Standard-of-Care Biomarkers						
MSI-H/dMMR	10–15% overall; 4–5% metastatic CRC	IHC, PCR, NGS	Prognostic biomarker; predictor of immunotherapy response; Lynch syndrome screening	Pembrolizumab, nivolumab, ipilimumab	1A	[1,9]
KRAS	40–55%	NGS, PCR	Predictor of anti-EGFR resistance	KRAS G12C-targeted therapies under clinical implementation	1A	[1,15,16,17]
NRAS	3–8%	NGS, PCR	Predictor of anti-EGFR resistance	No approved targeted therapy	1A	[1,18]
Extended RAS (KRAS/NRAS)		NGS, PCR	Guideline-supported predictor of resistance to anti-EGFR antibodies. Therapeutic implication: Restriction of cetuximab or panitumumab to RAS wild-type disease	Direct targeted options currently apply only to selected KRAS variants, not NRAS.	1A	[17,18]
BRAF V600E	8–15%	NGS, PCR	Adverse prognostic biomarker; therapeutic stratification	Encorafenib + cetuximab ± binimetinib	1A	[1,19]
HER2 Amplification	2–5%	IHC, ISH, NGS	Resistance mechanism to anti-EGFR therapy; predictive biomarker	Trastuzumab, tucatinib, trastuzumab deruxtecan	2A	[2,20]
NTRK Fusions	<1%	RNA-based NGS, pan-TRK IHC	Tumor-agnostic predictive biomarker	Larotrectinib, entrectinib	2A	[21,22]
Tier 2—Emerging Clinical Biomarkers						
Tumor Mutational Burden (TMB)	Variable	Comprehensive NGS	Potential predictor of immunotherapy benefit	Investigational	2B	[23]
POLE/POLD1 Mutations	<3%	NGS	Identification of ultramutated tumors; potential immunotherapy predictor	Investigational	2B	[24,25]
Circulating Tumor DNA (ctDNA)	Not prevalence-dependent	Liquid biopsy (NGS/ddPCR)	Minimal residual disease detection; recurrence monitoring; resistance assessment	Expanding clinical implementation	2B	[25,26]
DDR Alterations	5–15%	NGS panels	Potential predictor of immunotherapy responsiveness	PARP inhibitor-based strategies under investigation	2B	[26,27]
Tier 3—Future Precision Oncology Biomarkers						
Transcriptomic Signatures (CMS)	Applicable across CRC	RNA sequencing	Prognostic stratification; treatment-response prediction	Investigational	3	[26,27,28]
Microbiome-Derived Biomarkers	Emerging	Metagenomic sequencing	Prediction of immunotherapy response; host–tumor interaction profiling	Investigational	3	[29,30]
Epigenetic Biomarkers (SEPT9, methylation signatures)	Emerging	Methylation assays, NGS	Early detection and prognostic stratification	Investigational	3	[31]
Non-Coding RNAs (miRNAs, lncRNAs)	Emerging	RNA profiling	Prognostic and predictive biomarker development	Investigational	3	[32,33]
AI/Radiogenomic Biomarkers	Emerging	Digital pathology, CT/MRI radiomics	Non-invasive molecular prediction and treatment-response estimation	Future precision oncology applications	3	[34]

1A = Established standard of care; 2A = Clinically actionable in selected settings; 2B = Emerging/under clinical validation; 3 = Investigational.

### 3.5. Hereditary and Germline Predisposition to CRC

Although most CRCs are sporadic, a clinically important proportion arises in the setting of inherited cancer-predisposition syndromes. The distinction between somatic tumor alterations and germline pathogenic variants is essential because germline findings have implications not only for the affected patient but also for biologically related family members [33].

Lynch syndrome is the most common hereditary CRC syndrome and results from germline pathogenic variants involving *MLH1*, *MSH2*, *MSH6*, *PMS2*, or *EPCAM*. Tumor dMMR or MSI-H status may provide an initial indication of Lynch syndrome; however, sporadic MLH1 promoter hypermethylation is also a frequent cause of dMMR. Therefore, tumor testing should be integrated with *MLH1* methylation or *BRAF* assessment, clinical and family-history evaluation, genetic counseling, and germline testing when appropriate [34].

Adenomatous polyposis syndromes include familial adenomatous polyposis and attenuated familial adenomatous polyposis, most commonly caused by pathogenic *APC* variants, as well as *MUTYH*-associated polyposis, which follows an autosomal-recessive inheritance pattern and is generally associated with biallelic *MUTYH* variants. Other inherited conditions involving genes related to DNA proofreading, base-excision repair, or hamartomatous polyposis may also increase CRC risk.

Identification of a hereditary syndrome directly influences clinical management through intensified colonoscopic surveillance, screening for extracolonic malignancies, selection and timing of prophylactic or risk-reducing surgery, reproductive counseling, and cascade testing of relatives. Germline genetics should therefore be regarded as an integral component of the CRC genetic landscape rather than solely as an adjunct to somatic molecular profiling [35].

### 3.6. Mechanistic Integration of KRAS, NRAS, BRAF, and *HER2* Alterations

Under physiological conditions, ligand binding promotes EGFR dimerization and activation, followed by recruitment of intracellular adaptor proteins and conversion of RAS from its inactive GDP-bound form to the active GTP-bound state. Activated RAS subsequently stimulates RAF, MEK, and ERK, resulting in transcriptional programs that regulate cellular proliferation, differentiation, and survival. Parallel activation of the PI3K–AKT pathway contributes to cell survival, metabolism, and resistance to apoptosis.

Activating mutations in *KRAS* or *NRAS* impair normal GTP hydrolysis and maintain RAS in a constitutively active state. Consequently, downstream MAPK signaling becomes partially independent of extracellular EGFR activation. This mechanism explains why RAS-mutated tumors generally do not benefit from cetuximab or panitumumab despite pharmacological inhibition of the upstream receptor. Extended RAS testing therefore serves primarily as a negative predictive biomarker for anti-EGFR therapy. The negative association between *KRAS* or *NRAS* mutations and cetuximab benefit was demonstrated in large molecular analyses of patients with metastatic CRC [35].

*BRAF* functions immediately downstream of RAS. The V600E substitution produces constitutive *BRAF* kinase activation and persistent MEK–ERK signaling. In CRC, *BRAF* inhibition alone is insufficient because pathway suppression triggers rapid feedback activation of EGFR and reactivation of MAPK signaling. This feedback reactivation provides the biological rationale for combined *BRAF* and EGFR inhibition, validated in previously treated disease by BEACON CRC and subsequently extended to first-line treatment through BREAKWATER [36].

HER2, encoded by *ERBB2*, is a receptor tyrosine kinase capable of forming highly active homo- or heterodimers with other members of the ERBB receptor family. Amplification or overexpression of HER2 activates both the MAPK and PI3K–AKT pathways and may bypass dependence on EGFR signaling. HER2 amplification may, therefore, contribute to resistance to anti-EGFR treatment in selected *RAS/BRAF* wild-type tumors while simultaneously defining a subgroup susceptible to HER2-directed treatment. Clinical activity has been demonstrated with trastuzumab plus lapatinib in the HERACLES study and with tucatinib plus trastuzumab in MOUNTAINEER [20,37].

## 4. Clinically Actionable Biomarkers in CRC

The transition from conventional histopathological classification toward precision oncology has fundamentally transformed CRC management. Molecular biomarkers now play a central role in diagnosis, prognostic assessment, treatment selection, and therapeutic monitoring. Current international guidelines recommend assessment of MMR/MSI and extended RAS and *BRAF* status in appropriate CRC settings, while HER2 amplification and *NTRK* fusions are evaluated particularly in advanced disease when the results may guide molecularly matched treatment [38].

Among established CRC biomarkers, MSI-H/dMMR status is a clinically validated predictor of benefit from immune-checkpoint inhibition. More recently, rare genomic alterations such as HER2 amplification and *NTRK* fusions have expanded the therapeutic landscape of CRC, providing novel opportunities for targeted interventions.

### 4.1. Microsatellite Instability and Mismatch Repair Deficiency

Microsatellite instability (MSI) represents a cornerstone biomarker of precision oncology in CRC. MSI results from defective DNA mismatch repair (dMMR), typically involving loss of function of the *MLH1*, *MSH2*, *MSH6*, or *PMS2* genes. Approximately 10–15% of all CRCs and about 4–5% of metastatic CRCs exhibit MSI-H/dMMR. These tumors may arise through Lynch syndrome-associated germline alterations or through sporadic *MLH1* promoter hypermethylation [39].

The clinical significance of MSI-H tumors is largely related to their high mutational burden and increased neoantigen generation, which promote strong antitumor immune responses. Initially recognized as a prognostic biomarker, MSI-H status is now primarily valued as a predictive biomarker for immunotherapy. The introduction of immune checkpoint inhibitors (ICIs) has transformed the management of MSI-H/dMMR CRC, with studies consistently demonstrating superior outcomes compared with conventional chemotherapy. The KEYNOTE-177 trial established pembrolizumab as a first-line treatment option for metastatic MSI-H/dMMR CRC, confirming the pivotal role of MSI testing in therapeutic decision-making [40,41,42].

Several approaches are available for MSI assessment, including immunohistochemistry (IHC), polymerase chain reaction (PCR)-based assays, and next-generation sequencing (NGS). Validated NGS platforms may provide an efficient option when simultaneous assessment of MSI status and additional genomic alterations is clinically indicated, although IHC and PCR remain established methods for MMR/MSI evaluation [23].

Despite its established clinical value, MSI alone does not fully explain treatment-response heterogeneity. Although MSI-H/dMMR status is strongly predictive of benefit, not all tumors respond, and primary or acquired resistance may be influenced by diagnostic misclassification, impaired antigen presentation, immune exclusion, and additional genomic or microenvironmental factors. Consequently, current research is increasingly focused on integrated biomarker models that combine MSI with genomic, transcriptomic, and immune-related parameters [33,34,35].

#### MSI/dMMR in Metastatic CRC

In metastatic CRC, MSI-H/dMMR status is an established predictive biomarker for immune-checkpoint inhibition. These results established PD-1 blockade as a major first-line treatment option for this molecular subgroup [36].

More recently, the phase III CheckMate 8HW trial evaluated combined PD-1 and CTLA-4 inhibition. In the first-line comparison, nivolumab plus ipilimumab produced substantially longer progression-free survival than chemotherapy, with 24-month progression-free survival rates of 72% and 14%, respectively [37]. A subsequent randomized analysis across treatment lines also demonstrated superior progression-free survival with nivolumab plus ipilimumab compared with nivolumab monotherapy. These findings support dual immune-checkpoint blockade as an important treatment option for MSI-H/dMMR metastatic CRC, although treatment selection should also consider immune-related toxicity, comorbidities, treatment availability, and patient preference (Table 2).

**Table 2:** Representative Pivotal Studies Defining the Prognostic and Predictive Roles of MSI/dMMR in Colorectal Cancer.

Study	Clinical Setting and Population	MSI/dMMR Definition	MSI/dMMR Population	Principal Finding	Clinical Interpretation
Ribic et al., 2003 [29]	Retrospective analysis of patients with stage II–III colon cancer from randomized adjuvant studies	PCR-based microsatellite testing; MSI-H distinguished from MSI-L/MSS	Of 570 evaluable tumors, 95 (16.7%) were MSI-H	Fluorouracil-based adjuvant therapy benefited patients with MSS/MSI-L tumors but did not demonstrate benefit in MSI-H tumors	Established the prognostic and treatment-predictive importance of MSI in localized colon cancer and supported avoidance of fluoropyrimidine monotherapy in stage II MSI-H disease
KEYNOTE-177 [43]	Phase III first-line trial; 307 patients with unresectable or metastatic CRC	Centrally confirmed MSI-H or dMMR	All enrolled patients were MSI-H/dMMR	Median PFS was 16.5 months with pembrolizumab versus 8.2 months with chemotherapy; ORR was 43.8% versus 33.1%	Established MSI-H/dMMR as a predictive biomarker for first-line PD-1 blockade in metastatic CRC
CheckMate 8HW	Phase III first-line comparison in unresectable or metastatic CRC	Locally confirmed MSI-H and/or dMMR	All patients in the primary comparison were MSI-H/dMMR	Twenty-four-month PFS was 72% with nivolumab plus ipilimumab versus 14% with chemotherapy	Demonstrated substantial benefit from combined PD-1 and CTLA-4 blockade in metastatic MSI-H/dMMR CRC
NICHE-2	Prospective neoadjuvant study in locally advanced, non-metastatic colon cancer	dMMR confirmed before enrollment	111 patients included in the efficacy analysis	Pathological response occurred in 109/111 patients (98%); major pathological response in 95% and pathological complete response in 68%	Demonstrated marked sensitivity of localized dMMR colon cancer to short-course neoadjuvant immune-checkpoint blockade
Cercek et al., 2025 [44]	Prospective nonoperative-management study in locally advanced rectal cancer	dMMR confirmed before treatment	49 patients with rectal cancer completed dostarlimab treatment	All 49 patients achieved a clinical complete response and elected nonoperative management	Supports organ-preserving strategies in carefully selected dMMR rectal cancer, although long-term surveillance and broader validation remain essential

Abbreviations: dMMR, deficient mismatch repair; MSI, microsatellite instability; MSI-H, microsatellite instability-high; MSI-L, microsatellite instability-low; MSS, microsatellite stable; ORR, objective response rate; PFS, progression-free survival. MSI/dMMR was assessed using different platforms and eligibility definitions across studies. Results should therefore not be interpreted as a pooled MSI score or direct cross-trial comparison.

Nevertheless, MSI-H/dMMR status does not guarantee response. Primary resistance may reflect misclassification of MMR/MSI status, limited tumor immunogenicity, impaired antigen presentation, immune-exclusion mechanisms, or coexisting genomic and microenvironmental factors. Therefore, treatment failure should prompt verification of the molecular diagnosis and evaluation of alternative resistance mechanisms rather than an assumption that all MSI-H tumors share the same therapeutic sensitivity.

### 4.2. KRAS and NRAS Mutations: Predictors of Anti-EGFR Resistance and Emerging Therapeutic Targets

Mutations in the RAS gene family are among the most common molecular alterations in CRC and play a central role in therapeutic decision-making. *KRAS* mutations occur in approximately 40–55% of CRC cases, whereas *NRAS* mutations are identified in 3–8% of tumors. Both alterations result in constitutive activation of the MAPK signaling pathway, promoting tumor growth and reducing dependence on EGFR-mediated signaling [1].

The most important clinical implication of RAS mutations is their predictive value for resistance to anti-EGFR monoclonal antibodies. Multiple clinical trials have demonstrated that patients harboring *KRAS* or *NRAS* mutations derive minimal benefit from cetuximab or panitumumab therapy. Current guidelines recommend extended RAS testing before anti-EGFR treatment, restricting cetuximab or panitumumab to appropriately selected RAS wild-type metastatic CRC, restricting these therapies to patients with RAS wild-type tumors [45].

Most RAS-mutated metastatic CRCs are microsatellite stable, and RAS mutations exclude benefit from currently approved anti-EGFR antibodies. Although targeted options have expanded for selected variants such as *KRAS* G12C, many other RAS-mutated tumors continue to lack directly matched therapies [43,46,47,48,49].

*KRAS* G12C mutations, present in a small proportion of CRCs, can be targeted using *KRAS* G12C inhibitors, with greater clinical activity generally observed when these agents are combined with EGFR blockade [50,51].

Although *NRAS* mutations similarly predict resistance to anti-EGFR antibodies, no *NRAS*-specific targeted treatment has been established as a standard therapy in CRC [42].

Beyond their established role as predictive biomarkers, RAS mutations are increasingly being investigated through liquid biopsy technologies. ctDNA studies have described the disappearance of previously detectable RAS-mutant clones after withdrawal of anti-EGFR therapy, a phenomenon often termed NeoRAS conversion and investigated as a potential basis for anti-EGFR rechallenge [52,53].

### 4.3. BRAF V600E: From Adverse Prognostic Marker to First-Line Therapeutic Target

*BRAF* mutations are among the most clinically relevant molecular alterations in CRC because of their strong prognostic impact and increasing therapeutic actionability [54]. *BRAF* V600E identifies a clinically and biologically distinct subgroup of CRC characterized by aggressive disease behavior, frequent right-sided localization, and complex interactions with MSI and CpG island methylation. Its interpretation should therefore include assessment of MSI/dMMR status, particularly because *BRAF* V600E may accompany sporadic *MLH1* promoter methylation [51].

The phase III BEACON CRC trial established combined *BRAF* and EGFR inhibition for previously treated *BRAF* V600E-mutated metastatic CRC. Encorafenib plus cetuximab, with or without binimetinib, improved survival and response outcomes compared with irinotecan-based therapy plus cetuximab. The doublet of encorafenib and cetuximab subsequently became the principal targeted strategy in the previously treated setting [51].

The therapeutic role of *BRAF* inhibition has now moved into first-line treatment. In the phase III BREAKWATER trial, the addition of encorafenib and cetuximab to mFOLFOX6 resulted in significantly longer progression-free and overall survival compared to standard first-line treatment in patients with *BRAF* V600E-mutated metastatic CRC. These findings represent a major change from the previous treatment sequence, in which *BRAF*-targeted therapy was generally reserved for disease progression after at least one prior systemic regimen [36].

Resistance remains clinically relevant and may involve MAPK pathway reactivation, EGFR-mediated signaling, alternative RAS/RAF alterations, *MET* or PI3K pathway activation, and intratumoral clonal heterogeneity. Acquired resistance to *BRAF*/EGFR-directed therapy may involve MAPK pathway reactivation, emergent RAS/RAF alterations, EGFR-mediated signaling, *MET* amplification, PI3K-pathway alterations, and clonal heterogeneity [55,56,57].

### 4.4. HER2 Alterations in CRC: An Emerging Precision Oncology Target

HER2 amplification occurs in a small subset of CRCs, particularly among *RAS/BRAF* wild-type metastatic tumors. Although HER2 amplification is well established in breast and gastric cancers, its clinical relevance in CRC has only recently been recognized through advances in molecular profiling and targeted therapy development [58].

HER2 alterations occur in approximately 2–5% of CRC cases and are more frequently observed in metastatic, *RAS/BRAF* wild-type tumors. Activation of HER2 signaling promotes tumor growth through the MAPK and PI3K/AKT pathways and may contribute to resistance against anti-EGFR therapies. Consequently, HER2-positive CRC represents a distinct molecular subtype with important therapeutic implications [2].

One of the most significant clinical observations is the association between HER2 amplification and resistance to cetuximab or panitumumab. Some patients with *RAS/BRAF* wild-type tumors fail to respond to anti-EGFR therapy because of HER2-driven downstream signaling, highlighting the importance of comprehensive molecular testing [5].

The therapeutic landscape of HER2-positive CRC has evolved rapidly. Dual HER2 blockade with combinations such as trastuzumab plus pertuzumab or trastuzumab plus lapatinib has demonstrated meaningful clinical activity in metastatic disease. Tucatinib plus trastuzumab demonstrated clinically meaningful activity in MOUNTAINEER, while trastuzumab deruxtecan was evaluated in the DESTINY-CRC program [59].

Accurate identification of HER2-positive tumors relies on immunohistochemistry (IHC), fluorescence in situ hybridization (FISH), and increasingly on next-generation sequencing (NGS), which enables simultaneous assessment of multiple actionable biomarkers. As comprehensive genomic profiling becomes more widely adopted, early detection of HER2 alterations is expected to play an increasingly important role in precision oncology strategies [60].

Ongoing research is focused on optimizing HER2-targeted therapies, overcoming resistance mechanisms, and evaluating combinations with immunotherapy and novel antibody–drug conjugates [61].

### 4.5. NTRK Fusions: Rare Alterations with High Therapeutic Relevance

*NTRK* gene fusions represent rare but highly actionable molecular alterations in CRC. Although they occur in less than 1% of CRC cases, these genomic events have gained substantial clinical importance because they predict remarkable responses to selective TRK inhibitors. Unlike many conventional biomarkers, *NTRK* fusions are considered tumor-agnostic biomarkers, meaning that treatment eligibility is determined by the presence of the fusion rather than the tissue of origin [62,63].

The *NTRK* gene family comprises *NTRK1*, *NTRK2*, and *NTRK3*, which encode the TRKA, TRKB, and TRKC receptors, respectively. Chromosomal rearrangements involving these genes generate constitutively active fusion proteins that drive persistent oncogenic signaling through pathways such as MAPK and PI3K/AKT [58].

NTRK fusions are more frequently observed in specific molecular subgroups, including MSI-H/dMMR tumors and *RAS/BRAF* wild-type CRC. Despite their rarity, they define a clinically relevant subset of patients who may derive substantial benefit from targeted therapy. RNA-based NGS is particularly useful for confirming expressed NTRK fusions, while DNA-based NGS and pan-TRK IHC may serve as components of context-dependent testing algorithms [5].

The development of TRK inhibitors has transformed the management of *NTRK* fusion-positive cancers. Larotrectinib and entrectinib have demonstrated high response rates across *NTRK* fusion-positive solid tumors, although CRC-specific patient numbers remain limited. These findings highlight the importance of comprehensive molecular profiling and reinforce the shift toward biomarker-driven treatment selection [24,52].

Although resistance to first-generation TRK inhibitors may occur, next-generation agents such as selitrectinib and repotrectinib are being investigated to overcome acquired resistance mechanisms. As molecular diagnostics continue to evolve, improved detection of rare but highly actionable alterations such as *NTRK* fusions is expected to further expand precision oncology opportunities in CRC [53].

## 5. Emerging Biomarkers Beyond Standard Molecular Testing

Although MSI/dMMR, RAS, *BRAF*, HER2, and *NTRK* alterations constitute the foundation of molecular testing in CRC, these biomarkers do not fully capture the biological complexity of the disease. Significant heterogeneity persists among patients with apparently similar molecular profiles, resulting in substantial variability in treatment response, resistance development, and clinical outcomes.

Recent advances in next-generation sequencing, liquid biopsy technologies, transcriptomics, artificial intelligence, and multi-omics integration have identified a new generation of biomarkers capable of refining patient stratification and improving therapeutic decision-making. These emerging biomarkers may complement established molecular markers and contribute to a more comprehensive precision oncology framework.

### 5.1. Tumor Mutational Burden (TMB): A Context-Dependent and Controversial Biomarker

TMB refers to the number of somatic coding mutations detected per megabase of analyzed genomic sequence. Its potential predictive value in CRC remains context-dependent and controversial. A high number of mutations may increase neoantigen generation and theoretically enhance tumor recognition by the immune system. However, TMB is not a single standardized biological measurement. Its value varies according to sequencing platform, panel size, bioinformatic pipeline, germline filtering, variant inclusion criteria, tumor purity, and the threshold used to define a high result [20].

Interest in TMB as a treatment-selection biomarker increased following the tumor-agnostic evaluation of pembrolizumab in TMB-high solid tumors. A threshold of at least 10 mutations per megabase was used in the KEYNOTE-158 biomarker analysis. Nevertheless, that analysis evaluated selected non-colorectal tumor cohorts and did not establish that the same threshold possesses equivalent predictive value in CRC. Extrapolation of a universal numerical cutoff across tumor types may overlook major differences in tumor biology, neoantigen quality, immune contexture, and the mutational processes responsible for the elevated TMB [20,46].

In CRC, the biological cause of hypermutation appears more informative than the numerical TMB value alone. Most markedly hypermutated CRCs are associated with MSI-H/dMMR or pathogenic exonuclease-domain mutations in *POLE* or *POLD1*. These mechanisms generate immunogenic tumors that may respond to immune-checkpoint inhibition. By contrast, microsatellite-stable tumors that marginally exceed a threshold such as 10 mutations per megabase may not share the same immune phenotype. Passenger mutations, treatment-associated mutagenesis, assay variability, and pathogenic alterations unrelated to enhanced antigenicity may increase the reported TMB without producing meaningful immune sensitivity [64].

Recent CRC-specific evidence argues against using TMB-high status alone as a universal indication for immunotherapy in microsatellite-stable disease. In a retrospective genomic and clinical analysis of MSS/TMB-high metastatic CRC, pembrolizumab was associated with substantially shorter treatment duration and overall survival than observed in MSI-H/TMB-high disease. Within the MSS/TMB-high subgroup, outcomes with pembrolizumab were also inferior to those observed with trifluridine/tipiracil in later-line treatment. Although retrospective comparisons are susceptible to selection bias and residual confounding, these findings indicate that a numerical TMB-high result does not reliably identify an immunotherapy-sensitive MSS CRC population [65].

TMB may still provide complementary information within MSI-H/dMMR CRC, where higher values have been associated in some cohorts with improved outcomes following immune-checkpoint inhibition. However, no prospectively validated CRC-specific threshold currently justifies selecting or excluding an established immunotherapy solely on the basis of TMB within the MSI-H population. Similarly, pathogenic *POLE/POLD1* mutations should be interpreted according to the affected domain, variant pathogenicity, and characteristic mutational signature rather than inferred from an elevated TMB value alone [66].

Therefore, TMB should currently be regarded as a context-dependent biomarker whose interpretation requires integration with MSI/MMR status, *POLE/POLD1* alterations, the underlying mutational signature, assay characteristics, and the clinical setting. For routine CRC management, MSI-H/dMMR remains the validated immunotherapy biomarker, whereas isolated TMB-high status in microsatellite-stable disease should be considered investigational and should preferably be evaluated within clinical trials.

### 5.2. Pathogenic POLE/POLD1 Proofreading-Domain Alterations

Mutations affecting the exonuclease domains of DNA polymerases epsilon (*POLE*) and delta (*POLD1*) represent rare but highly significant genomic alterations in CRC. These pathogenic exonuclease-domain or proofreading-deficient alterations impair DNA proofreading mechanisms, resulting in an ultramutated phenotype characterized by exceptionally high mutation burdens [24].

*POLE/POLD*1-mutated tumors frequently exhibit mutational loads exceeding those observed in MSI-H cancers and often generate substantial neoantigen repertoires capable of stimulating robust immune responses. Pathogenic proofreading-domain POLE/POLD1 alterations may identify a rare subgroup of ultramutated tumors with increased immunogenicity and potential sensitivity to immune-checkpoint inhibition, including selected microsatellite-stable cancers [67,68].

From a clinical perspective, *POLE/POLD1* mutations are particularly important because they identify a subset of patients who may benefit from immunotherapy despite lacking traditional MSI-H/dMMR biomarkers. This observation highlights the limitations of relying exclusively on MSI testing and supports broader genomic profiling approaches [68].

Although rare, pathogenic proofreading-deficient *POLE/POLD1* alterations represent promising predictive biomarkers requiring further prospective validation.

### 5.3. Circulating Tumor DNA (ctDNA): From Molecular Residual Disease Detection to Treatment-Guided Applications

ctDNA represents the tumor-derived fraction of cell-free DNA detected in plasma. Its short half-life and capacity to reflect genomic alterations originating from multiple tumor sites make ctDNA an attractive biomarker for assessing molecular residual disease, monitoring treatment response, identifying acquired resistance, and characterizing clonal evolution. However, the clinical interpretation of ctDNA depends on the disease setting, assay design, sampling time, tumor burden, and biological shedding characteristics [59].

Current ctDNA assays can be broadly divided into tumor-informed and tumor-agnostic approaches. Tumor-informed assays first identify patient-specific alterations in resected tumor tissue and subsequently track these variants in plasma. This strategy can provide high specificity for molecular residual disease but requires adequate tumor tissue and additional assay-development time. Tumor-agnostic approaches analyze predefined genomic or epigenomic features directly in plasma and may be operationally faster, although their sensitivity and specificity vary according to panel composition and analytical depth [69,70].

The most extensively studied application in localized CRC is postoperative molecular residual disease assessment. Across prospective cohorts, detectable postoperative ctDNA is consistently associated with a markedly increased recurrence risk. In the large GALAXY observational cohort, postoperative and longitudinal ctDNA positivity strongly predicted disease-free and overall survival. Sustained ctDNA clearance during adjuvant chemotherapy was associated with substantially better outcomes than transient clearance or persistent positivity, supporting ctDNA dynamics as a potential indicator of treatment efficacy [71].

Prognostic association, however, is not equivalent to treatment-guiding utility. In the randomized DYNAMIC trial, a ctDNA-guided strategy reduced adjuvant chemotherapy use without compromising recurrence-free survival in stage II colon cancer. Nevertheless, this result should not be generalized automatically to all stages, risk groups, assays, or treatment decisions. Ongoing randomized studies are evaluating whether ctDNA can safely guide treatment de-escalation in molecularly negative patients and improve outcomes through escalation in molecularly positive patients [72].

In metastatic CRC, ctDNA can be used to monitor treatment response and identify acquired resistance alterations involving *RAS*, *BRAF*, EGFR, *ERBB2*, *MET*, and other pathways. In CHRONOS, plasma RAS/BRAF/EGFR screening was used to select patients for panitumumab rechallenge; the objective response rate was 30%, and disease control was achieved in 63%. These findings indicate that ctDNA can contribute not only to prognosis but also to real-time therapeutic selection in selected metastatic settings [73].

Several limitations currently prevent uniform implementation. A negative plasma result does not necessarily exclude residual or progressive disease because ctDNA shedding is influenced by tumor volume, metastatic site, vascularity, treatment exposure, and assay sensitivity. Peritoneal and selected pulmonary recurrences may be associated with relatively limited plasma shedding. Pre-analytical factors, the timing of sampling after surgery or treatment, clonal hematopoiesis, differences between tumor-informed and fixed-panel assays, and the absence of standardized positivity thresholds further complicate interpretation [74].

ctDNA should therefore be described as an established prognostic biomarker for postoperative recurrence risk and an emerging treatment-guidance tool rather than as a universally implemented replacement for tissue analysis, imaging, or standard clinicopathological assessment. Its greatest clinical value is likely to occur when testing is linked to a predefined therapeutic decision and evaluated in prospective biomarker-guided trials [75].

### 5.4. Transcriptomic and Immune Signatures

While genomic alterations provide important information regarding tumor biology, they do not fully capture the dynamic interactions occurring within the tumor microenvironment. Consequently, transcriptomic profiling provides complementary functional information on tumor biology and has contributed to the identification of biologically distinct CRC subtypes [76].

The Consensus Molecular Subtype framework classifies CRC into four principal transcriptomic groups: CMS1, CMS2, CMS3, and CMS4 [25]. Each subtype exhibits distinct biological characteristics, prognostic profiles, and therapeutic vulnerabilities. CMS1 tumors are enriched for MSI-H status and immune activation and are therefore biologically associated with an immunogenic phenotype; however, CMS classification is not currently used as an independent routine biomarker for immunotherapy selection. CMS2 tumors are characterized by Wnt and MYC pathway activation, whereas CMS3 tumors display metabolic dysregulation. CMS4 tumors demonstrate stromal activation, angiogenesis, immune suppression, and generally poorer clinical outcomes [77].

Beyond CMS classification, increasing attention has been directed toward immune-related transcriptomic signatures capable of predicting immunotherapy response. Immune-related transcriptomic signatures commonly incorporate expression patterns associated with cytotoxic lymphocyte activity, interferon signaling, antigen processing, and immune-checkpoint pathways [13].

Immune and microenvironmental factors may contribute to heterogeneity in response among MSI-H/dMMR tumors. Tumor microenvironment-aware transcriptomic models have shown encouraging results in predicting MSI status and immunotherapy responsiveness, highlighting the growing importance of transcriptomic biomarkers in precision oncology [69,78].

The integration of transcriptomic profiling with genomic and clinical data is expected to further improve risk stratification and therapeutic decision-making in CRC.

### 5.5. Artificial Intelligence (AI) and Radiogenomics in CRC

AI is rapidly transforming oncology by enabling the analysis of complex datasets that exceed human interpretative capacity. In CRC, AI is being investigated across digital pathology, imaging analysis, molecular prediction, and prognostic modeling [70,79].

One of the most promising developments involves AI-assisted prediction of molecular biomarkers directly from routine histopathological images. Deep learning algorithms have demonstrated the ability to identify MSI-H tumors with high accuracy using digitized hematoxylin and eosin (H&E) slides, potentially supporting prescreening or prioritization for confirmatory molecular testing. Several studies have reported encouraging performance for AI-based MSI prescreening from routine histology slides [33,79].

Similarly, radiogenomics combines imaging data with molecular information to predict genomic alterations non-invasively. Radiomic models have also been investigated for predicting treatment response in dMMR/MSI-H CRC, including interpretable imaging-based models developed in the neoadjuvant immunotherapy setting [80].

Transcriptome-based computational models have been developed to predict MSI status and characterize tumor-microenvironment-related molecular patterns in CRC. Such systems may support individualized treatment selection, identify patients likely to benefit from targeted therapies or immunotherapy, and facilitate adaptive treatment strategies throughout disease progression [81].

Across oncology, radiogenomic approaches face challenges related to data standardization, external validation, interpretability, and regulatory implementation; these limitations also apply to emerging CRC applications [82].

Collectively, emerging biomarkers such as DDR alterations, transcriptomic signatures, and AI-derived predictive models illustrate the ongoing evolution of CRC management from single-gene testing toward integrated multi-omics precision medicine. These innovations have the potential to substantially improve patient stratification, optimize treatment selection, and enhance clinical outcomes in the coming years.

### 5.6. Microbiome-Derived Biomarkers: Expanding the Precision Oncology Landscape

The gut microbiota has been associated with CRC development, progression, immune regulation, and treatment response, although causal relationships and clinically validated biomarker applications remain incompletely established. Beyond genetic and transcriptomic alterations, microbial signatures are increasingly being investigated as potential biomarkers capable of complementing conventional molecular profiling and improving patient stratification [76,77].

Among the microorganisms most consistently associated with CRC, *Fusobacterium nucleatum* has emerged as a particularly relevant candidate biomarker. Numerous studies have demonstrated increased abundance of *F. nucleatum* in colorectal tumors, where it has been linked to enhanced tumor proliferation, immune evasion, metastatic potential, and poorer clinical outcomes [83]. Elevated intratumoral *F. nucleatum* abundance has been associated with MSI/MMR-related and epigenetic features in selected CRC cohorts [84].

Beyond individual bacterial species, growing evidence indicates that distinct microbial community profiles may predict treatment response. Across several malignancies, gut microbiome composition has been associated with response to immune-checkpoint inhibition; however, CRC-specific predictive signatures remain insufficiently validated [85,86].

The emergence of metagenomic sequencing and multi-omics technologies has facilitated the identification of complex microbiome signatures that may complement established biomarkers such as MSI, tumor mutational burden (TMB), and circulating tumor DNA (ctDNA). Future precision oncology models may therefore integrate genomic, transcriptomic, immune, and microbiome-derived information into comprehensive predictive frameworks capable of providing a more complete representation of tumor biology [86,87].

Although microbiome-based biomarkers remain largely investigational, their growing association with CRC pathogenesis, prognosis, and treatment response highlights their potential role as an important component of next-generation precision oncology strategies. Further prospective studies and standardization of microbiome assessment methodologies will be essential before widespread clinical implementation can be achieved.

### 5.7. Epigenetic Biomarkers and Non-Coding RNAs

Epigenetic alterations, including DNA methylation, histone modification, and regulation by non-coding RNAs, contribute to CRC development and molecular heterogeneity. Unlike genomic mutations, epigenetic changes modify gene expression without altering the underlying DNA sequence and include DNA methylation, histone modifications, and regulation by non-coding RNAs. These mechanisms influence tumor initiation, progression, metastasis, and therapeutic responsiveness, making them attractive candidates for biomarker development [88,89].

One of the most extensively studied epigenetic mechanisms in CRC is DNA methylation, particularly within CpG islands located in gene promoter regions. Aberrant promoter hypermethylation can lead to silencing of tumor suppressor genes, while global hypomethylation contributes to genomic instability. The CpG Island Methylator Phenotype (CIMP) represents a distinct molecular subtype of CRC characterized by widespread promoter hypermethylation and frequent associations with *BRAF* mutations and microsatellite instability [12,13].

Among clinically relevant methylation-based biomarkers, SEPT9 promoter methylation has gained particular attention. Circulating methylated SEPT9 DNA has been evaluated as a blood-based CRC detection assay, although it does not replace colonoscopy or established stool-based screening methods [90].

In parallel, increasing interest has focused on microRNAs (miRNAs) and long non-coding RNAs (lncRNAs), which regulate gene expression at transcriptional and post-transcriptional levels. Several miRNAs, including miR-21, miR-92a, and miR-135b, have been associated with tumor progression, metastasis, and prognosis, while dysregulated lncRNAs such as HOTAIR, MALAT1, and CCAT1 have been implicated in CRC proliferation, invasion, and treatment resistance [29]. Several miRNAs and lncRNAs have been associated with CRC progression, metastasis, and treatment resistance in preclinical and retrospective studies. However, heterogeneity in assay platforms, normalization methods, specimen types, and validation cohorts currently limits routine clinical implementation [91].

Recent advances in methylome profiling and next-generation sequencing technologies have enabled comprehensive characterization of epigenetic landscapes across CRC subtypes. These approaches may identify novel biomarkers capable of improving early detection, prognostic stratification, and therapeutic selection. Although most epigenetic and non-coding RNA biomarkers remain investigational, their integration with genomic, transcriptomic, and immune-based markers may contribute to the development of more comprehensive precision oncology models in CRC [92].

In summary, epigenetic alterations and non-coding RNAs represent a rapidly evolving area of CRC research, offering promising opportunities for non-invasive diagnostics, risk stratification, and future biomarker-guided therapeutic strategies.

## 6. Toward a Clinical Actionability Framework in CRC

The rapid expansion of molecular diagnostics has transformed CRC from a malignancy classified primarily by anatomical and histopathological characteristics into one increasingly defined by its molecular profile. However, the growing number of available biomarkers presents a practical challenge for clinicians: not all molecular alterations possess the same degree of clinical relevance, therapeutic actionability, or level of supporting evidence [83].

Current clinical practice often relies on a collection of individual biomarkers evaluated independently. While this approach has significantly improved patient management, it may not adequately reflect the three practical categories nature of molecular decision-making in precision oncology. Consequently, there is a need for an integrated framework that organizes biomarkers according to their current clinical utility, therapeutic implications, and future translational potential [6,92,93].

Based on the reviewed evidence, we use a practical CRC-oriented clinical implementation framework that organizes biomarker domains according to their current role in care and their level of clinical maturity.

### 6.1. Tier 1: Established Standard-of-Care Biomarkers

The first tier includes biomarkers that are currently incorporated into international clinical guidelines and directly influence routine therapeutic decisions. These biomarkers possess robust clinical validation and are routinely assessed in contemporary CRC management.

### 6.2. Tier 2: Emerging Clinical Biomarkers

The second tier comprises biomarkers supported by growing clinical evidence but not yet universally incorporated into routine treatment algorithms. These biomarkers have demonstrated promising predictive or prognostic value and are likely to play an increasingly important role in future clinical practice [94,95].

#### 6.2.1. Tumor Mutational Burden (TMB)

TMB may provide complementary biological information, but its predictive interpretation in CRC depends on MSI/MMR status, *POLE/POLD1* alterations, assay methodology, and the underlying mutational process. Isolated TMB-high status in MSS CRC remains investigational. Its integration into comprehensive genomic profiling platforms has facilitated broader clinical investigation [96].

#### 6.2.2. POLE/POLD1 Mutations

Although uncommon, *POLE/POLD1* alterations identify tumors with ultramutated phenotypes and potential sensitivity in selected pathogenic proofreading-deficient tumors to immune checkpoint inhibitors. These mutations may become important biomarkers for immunotherapy selection beyond MSI-H disease [97].

#### 6.2.3. Circulating Tumor DNA (ctDNA)

ctDNA has strong prognostic value for postoperative recurrence risk and is under prospective evaluation as a treatment-guidance tool in CRC. As assay sensitivity improves, ctDNA may become a routine component of longitudinal disease management [98].

#### 6.2.4. DNA Damage Repair (DDR) Alterations

DDR alterations remain investigational in CRC. Although biological rationale exists for immunotherapy and synthetic-lethality-based strategies, no broadly validated CRC-specific treatment-selection role has been established [99].

Although additional validation is required, Tier 2 biomarkers are rapidly approaching clinical implementation and are likely to become integral components of future molecular testing algorithms.

### 6.3. Tier 3: Future Precision Oncology Biomarkers

The third tier includes emerging technologies and molecular approaches that remain primarily investigational but possess substantial potential to reshape precision oncology over the coming decade [100,101].

#### 6.3.1. Transcriptomic Signatures

Gene expression-based classification systems provide functional information that complements genomic testing. Transcriptomic biomarkers may improve prediction of treatment response, prognosis, and tumor behavior beyond traditional mutation-based approaches [81,88,92].

#### 6.3.2. Immune Microenvironment Profiling

The composition and functional state of the tumor immune microenvironment influence responsiveness to immunotherapy and may explain clinical heterogeneity among tumors sharing similar genomic profiles [102,103].

#### 6.3.3. Artificial Intelligence-Based Biomarkers

AI-driven pathology and computational biomarker discovery have demonstrated encouraging results for MSI prediction, molecular classification, and treatment-response estimation. AI-based biomarkers have shown encouraging retrospective performance for tasks such as MSI prescreening and molecular classification, but prospective workflow validation and health-economic evaluation remain necessary [33,79].

Table 3 summarizes selected emerging molecular biomarkers and technology-enabled approaches according to their current level of development, potential clinical applications, and principal barriers to implementation. The categories are not intended to predict a fixed timeline for clinical adoption. Rather, they distinguish biomarkers undergoing advanced clinical validation from approaches that remain predominantly translational or hypothesis-generating. Importantly, analytical detectability should not be considered equivalent to demonstrated clinical utility, and routine implementation requires prospective evidence that biomarker-guided decisions improve patient outcomes.

#### 6.3.4. Radiogenomics

Radiomic and radiogenomic approaches have been investigated for non-invasive prediction of MSI status, treatment response, and tumor heterogeneity in CRC; however, current evidence remains predominantly retrospective and exploratory [80].

Although currently investigational, these biomarkers represent the frontier of precision oncology and may fundamentally redefine molecular classification systems in CRC.

**Figure 2:**
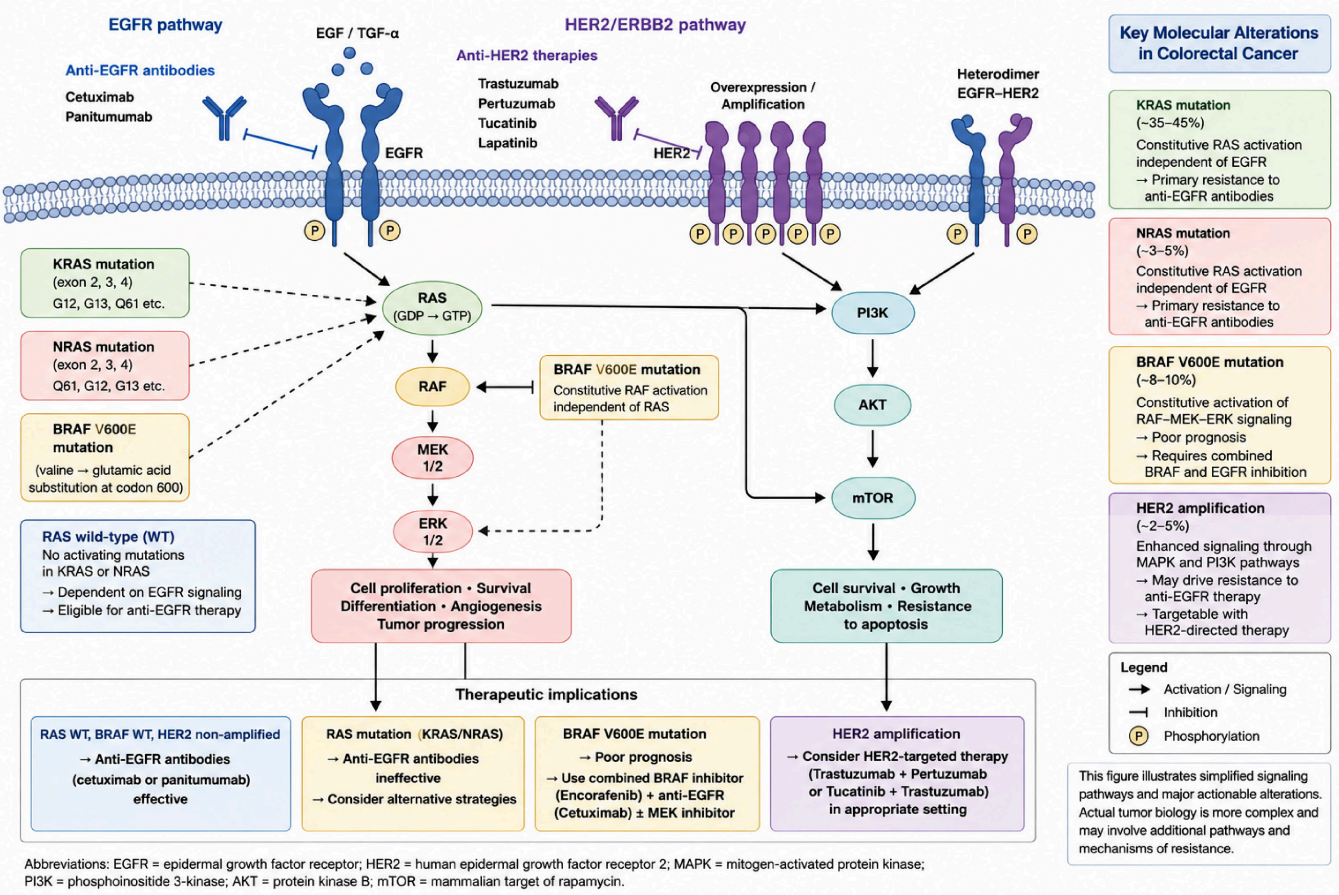
Mechanistic effects and therapeutic implications of *KRAS*, *NRAS*, *BRAF*, and HER2 alterations in colorectal cancer. Activation of EGFR or HER2 stimulates the RAS–RAF–MEK–ERK and PI3K–AKT–mTOR pathways, promoting cellular proliferation, survival, and tumor progression. Activating *KRAS* or *NRAS* mutations maintain RAS signaling independently of upstream EGFR activation and predict resistance to anti-EGFR antibodies. *BRAF* V600E constitutively activates downstream MAPK signaling; feedback reactivation of EGFR explains the requirement for combined *BRAF* and EGFR inhibition. HER2 amplification enhances MAPK and PI3K–AKT signaling, may provide an alternative mechanism of resistance to EGFR inhibition, and defines a subgroup potentially susceptible to HER2-directed therapies. Specific HER2-directed combinations and regulatory indications vary according to treatment setting and jurisdiction.

The growing complexity of molecular profiling in CRC necessitates a structured approach for biomarker prioritization [104]. Figure 2 illustrates the principal signaling relationships and therapeutic implications of *KRAS*, *NRAS*, *BRAF*, and HER2 alterations in CRC.

### 6.4. From Single Biomarkers to Integrated Precision Oncology

The evolution of CRC management reflects a broader transition occurring throughout oncology. Historically, treatment decisions were based primarily on anatomical staging and isolated molecular alterations. Contemporary precision oncology increasingly considers interactions among molecular alterations, tumor context, and temporal evolution rather than relying on single biomarkers alone [105].

Accordingly, the future of CRC management will likely depend on integrated molecular profiling rather than reliance on individual biomarkers. Comprehensive genomic profiling, transcriptomic analysis, liquid-biopsy monitoring, and AI-assisted interpretation are being investigated as complementary components of future integrated decision-support models [92,106].

The Clinical Actionability Framework proposed in this review provides a conceptual structure for organizing this rapidly expanding body of molecular information. By distinguishing established, emerging, and future biomarkers, this model may facilitate clinical interpretation, guide research priorities, and support the continued evolution of precision oncology in CRC [107].

**Figure 3:**
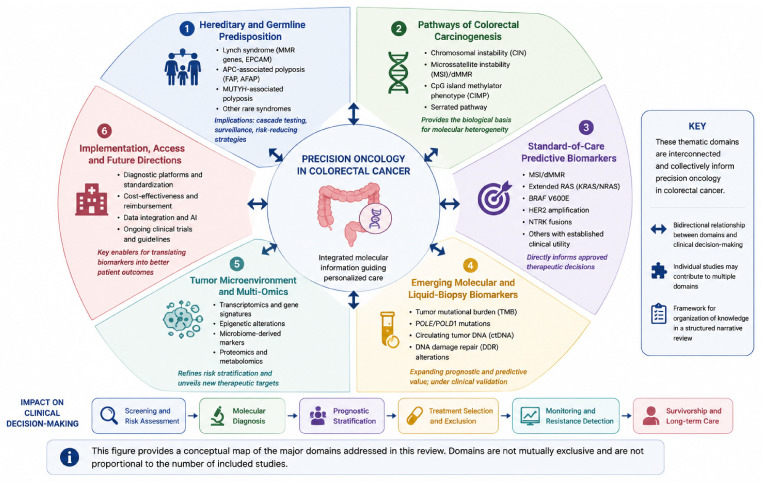
Thematic domains covered in the review and their relationship to clinical decision-making in CRC. The domains are not mutually exclusive, and the figure does not represent the numerical distribution of the included publications.

Figure 3 provides a conceptual overview of the principal thematic domains addressed in this review and illustrates how these domains contribute to clinical decision-making in CRC.

## 7. Challenges, Implementation Barriers, and Research Priorities in CRC Precision Oncology

The expanding molecular characterization of CRC has created important opportunities for hereditary-risk assessment, prognostic stratification, targeted treatment selection, minimal residual disease detection, and longitudinal monitoring of therapeutic resistance. However, the identification of a potentially relevant biomarker does not automatically translate into improved patient outcomes. Successful implementation requires analytical validity, biological representativeness, demonstrated clinical utility, timely access to testing and matched therapies, sustainable reimbursement, and integration into multidisciplinary decision-making. Accordingly, the next phase of precision oncology in CRC should focus not only on discovering additional biomarkers but also on determining how reliably, equitably, and cost-effectively existing and emerging biomarkers can be incorporated into real-world care [97,107,108,109].

### 7.1. Analytical Validity and Platform Standardization

The clinical interpretation of molecular biomarkers depends fundamentally on the analytical reliability of the testing platform. For mismatch repair and microsatellite instability assessment, immunohistochemistry (IHC), polymerase chain reaction (PCR), and next-generation sequencing (NGS) evaluate related but not identical biological features. IHC assesses the expression of the principal mismatch repair proteins MLH1, PMS2, MSH2, and MSH6 and may indicate which component of the MMR pathway is affected. PCR-based assays assess length variation in selected microsatellite loci, whereas validated NGS approaches infer MSI status from a larger set of genomic regions and may simultaneously identify tumor mutational burden and other clinically relevant alterations. Consequently, agreement among these methods is generally high, but complete interchangeability should not be assumed. Current pathology guidance emphasizes that each method must be validated for its intended clinical use and that assay selection should reflect specimen characteristics, laboratory expertise, and the therapeutic question being addressed [98]. Current pathology guidance emphasizes that each method should be analytically validated for its intended clinical use and that assay selection should reflect specimen characteristics, laboratory expertise, and the clinical question being addressed.

Discordant results may arise from both technical and biological factors. Although concordance between validated MMR-IHC and molecular MSI assays is generally high, discordant results may occur and can require pathological review, repeat testing, or confirmation using an orthogonal method. Potential explanations include insufficient tumor content, DNA degradation, prolonged or inadequate fixation, extensive necrosis or mucin, limited biopsy material, borderline instability patterns, subclonal loss of MMR protein expression, and assay-specific analytical thresholds. Potential causes include inadequate tumor content, fixation-related artifacts, DNA degradation, limited tissue, heterogeneous or subclonal protein loss, borderline molecular findings, and pathogenic variants that preserve antigenicity despite impaired MMR function [110].

In addition, some pathogenic missense variants may produce an antigenically detectable but functionally impaired protein, leading to retained IHC staining despite an abnormal molecular phenotype. Conversely, equivocal or focal IHC loss may not always correspond to MSI-H when assessed using a molecular assay [111].

Tumor cellularity is particularly important for molecular assays because a low proportion of neoplastic cells may dilute a mutant or unstable signal below the assay’s limit of detection. The minimum acceptable tumor fraction is platform-dependent and should therefore be specified in the laboratory validation documentation rather than inferred from a universal cutoff. Pathologist-guided selection and, where appropriate, macrodissection of the tumor-rich area are essential components of quality assurance. Reports should document specimen type, estimated tumor content, assay limitations, quality-control metrics, and the genomic regions or microsatellite loci evaluated [110].

A structured approach should be applied when IHC, PCR, and NGS results are discordant. This should include reassessment of tumor morphology and cellularity, review of internal positive controls, repetition of the original assay when technically justified, and confirmation using an orthogonal method. Evaluation of a different tumor block or metastatic specimen may be informative when intratumoral heterogeneity is suspected. When the pattern raises the possibility of Lynch syndrome, tumor findings should be integrated with *MLH1* promoter methylation analysis, *BRAF* testing where appropriate, clinical and family history, genetic counseling, and germline testing. Discordant results should not be resolved solely by automatically privileging one platform over another; instead, the final interpretation should reflect the combined pathological, molecular, and clinical evidence.

Standardization therefore requires more than the use of a common technology. Laboratories should participate in external quality-assurance programs, apply validated positive and negative controls, define platform-specific detection thresholds, and report assay failure rates and indeterminate findings. Harmonized reporting is required for MMR/MSI results and assay limitations [110]. Similar standardization challenges also affect TMB, ctDNA positivity, and variant classification, although the relevant thresholds and reporting standards differ across biomarker classes.

### 7.2. Biological Heterogeneity and Acquired Resistance

CRC is an evolutionary disease characterized by interpatient, intratumoral, spatial, and temporal heterogeneity. Many early driver alterations are shared between the primary tumor and metastatic lesions, and high concordance has been reported for several clinically established biomarkers, particularly common RAS and *BRAF* alterations. Nevertheless, broader genomic profiling may reveal differences in subclonal mutations, copy-number changes, tumor mutational burden, and resistance-associated alterations. Studies of matched primary and metastatic CRC specimens have generally reported high concordance for common truncal alterations such as RAS and *BRAF*, while broader genomic analyses may identify discordance in subclonal mutations, copy-number alterations, and resistance-associated changes. Reported concordance varies according to the genes examined, sequencing depth, metastatic site, timing of specimen collection, and previous treatment exposure [111].

This distinction is clinically important. High concordance for common truncal drivers does not imply that an archival primary-tumor specimen fully represents the molecular composition of all subsequent metastases. Minor resistant subclones may be absent from the sampled area, present below the analytical detection threshold, or selected during systemic treatment. In addition, metastatic lesions arising in different organs may evolve under distinct microenvironmental pressures. Therefore, a molecular result obtained at diagnosis represents a time- and site-specific sample rather than a permanent description of the patient’s disease.

Therapeutic exposure further increases temporal heterogeneity. Anti-EGFR treatment may select resistant clones harboring *RAS*, *BRAF*, EGFR extracellular-domain, *MET*, or *ERBB2* alterations. Circulating tumor DNA studies have shown that such resistant populations can emerge during treatment and decline after treatment withdrawal, providing a biological rationale for molecularly selected anti-EGFR rechallenge. Similar evolutionary processes contribute to resistance to *BRAF*-targeted combinations, HER2-directed therapy, *KRAS* G12C inhibition, and immune-checkpoint blockade.

These findings expose the limitations of a single tissue biopsy performed at one disease stage. Repeat tissue biopsy should be considered when the result is likely to alter treatment and when an accessible lesion can be sampled safely. Liquid biopsy may provide a complementary method for capturing alterations released from multiple metastatic sites and for monitoring clonal dynamics over time. However, a negative ctDNA result cannot always exclude an alteration because circulating DNA shedding varies according to tumor volume, anatomical distribution, treatment exposure, assay sensitivity, and blood-sampling conditions. Tissue and plasma testing should be considered complementary. Tissue analysis provides histopathological context and may detect alterations in tumors with limited plasma shedding, whereas ctDNA may capture spatially distributed and temporally evolving clones. A negative plasma result should not be interpreted as excluding an actionable alteration when clinical suspicion remains high [111,112,113,114,115].

Longitudinal molecular assessment is most clinically meaningful when it is linked to a predefined decision. Examples include confirming acquired resistance, selecting patients for anti-EGFR rechallenge, identifying postoperative molecular residual disease, or determining eligibility for a biomarker-directed clinical trial. Repeated testing without a clear therapeutic implication may increase cost and generate uncertain findings without improving patient care.

### 7.3. Clinical Utility, Access, and Reimbursement

Analytical actionability should be distinguished from clinical accessibility. A genomic alteration may be detectable and theoretically targetable, yet still fail to influence treatment because the result becomes available too late, the recommended drug is not reimbursed, the relevant trial is geographically inaccessible, or the patient is not clinically eligible for treatment. Therefore, the success of precision oncology should not be measured only by the proportion of tumors with potentially actionable alterations.

The direct price of a molecular assay represents only one component of the total diagnostic pathway. Additional costs may include retrieval and pathological review of tissue, macrodissection, DNA or RNA extraction, repeat testing after assay failure, confirmatory IHC or in situ hybridization, germline confirmation, genetic counseling, bioinformatic analysis, data storage, clinical interpretation, repeat biopsy, and additional consultations. Delays caused by sequential single-gene testing may also carry indirect costs if systemic treatment is initiated before a complete molecular profile is available [116,117].

Panel-based NGS may reduce tissue consumption and consolidate the evaluation of multiple biomarkers into a single workflow. Its economic value is nevertheless context-dependent. It is more likely to be favorable when several alterations must be tested simultaneously, when the result is available before treatment selection, and when patients can access matched therapies or clinical trials. Conversely, very broad panels may generate variants of uncertain significance, incidental germline findings, and theoretically actionable alterations without an available intervention. Real-world studies have demonstrated a substantial gap between the proportion of tumors with potentially actionable alterations and the smaller proportion of patients who ultimately receive matched therapy [107].

Turnaround time should be treated as a clinical quality indicator. Laboratories and oncology services should report the interval from test request to specimen receipt, from specimen receipt to validated molecular report, and from report issuance to multidisciplinary treatment decision. A comprehensive assay that requires several weeks may be less clinically useful than a focused assay delivered before first-line treatment, particularly in patients with rapidly progressive disease.

Bioinformatic capacity is another frequently underestimated requirement. NGS implementation requires validated pipelines, secure data storage, variant annotation, periodic knowledge-base updates, quality monitoring, and appropriately trained personnel. Smaller institutions may have access to sequencing technology but lack the expertise required to interpret complex alterations or distinguish clinically validated biomarkers from exploratory findings.

Molecular tumor boards can help bridge this interpretive gap by integrating pathology, molecular genetics, medical oncology, surgery, radiation oncology, genetic counseling, bioinformatics, and pharmacology. Their role should extend beyond producing a theoretical treatment recommendation. Relevant implementation outcomes include whether the recommendation was delivered before treatment selection, whether the suggested drug or trial was accessible, whether the patient received the intervention, and whether the recommendation changed clinical management.

Reimbursement frameworks should similarly account for the full care pathway. Funding a test without providing access to confirmatory testing, genetic counseling, matched therapy, or trial referral may create molecular information without corresponding clinical benefit. Cost-effectiveness analyses should therefore be conducted within specific healthcare systems and should include downstream treatment costs, avoided ineffective therapy, adverse events, repeat procedures, patient travel, and budget impact, rather than relying only on the purchase price of the assay.

### 7.4. Evidence Generation and Biomarker-Guided Clinical Trials

The most important transition in CRC biomarker research is from demonstrating prognostic association to proving that biomarker-guided intervention improves patient outcomes. Several contemporary trials illustrate this shift (Table 4).

The randomized DYNAMIC trial evaluated postoperative ctDNA-guided adjuvant treatment in stage II colon cancer. The ctDNA-guided strategy reduced the proportion of patients receiving adjuvant chemotherapy without compromising recurrence-free survival compared with conventional clinicopathological management. Mature follow-up subsequently showed similar five-year recurrence-free survival—approximately 88% in the ctDNA-guided group and 87% in the standard-management group—and similar five-year overall survival. These findings support the clinical potential of postoperative ctDNA, while also emphasizing that assay timing, sensitivity, management of ctDNA-positive disease, and applicability to different risk groups require continued evaluation.

CIRCULATE-US/NRG-GI008 (NCT05174169) extends this concept to patients with high-risk stage II and stage III colon cancer. The phase II/III study evaluates whether postoperative ctDNA can guide treatment de-escalation in molecularly negative patients and treatment escalation in molecularly positive patients. Its design directly addresses a key limitation of observational ctDNA studies by testing whether acting on the biomarker result improves clinically relevant outcomes rather than merely predicting recurrence [112].

The NICHE program, registered under NCT03026140, has evaluated short-course neoadjuvant immune-checkpoint blockade in non-metastatic colon cancer. In patients with locally advanced dMMR colon cancer, neoadjuvant nivolumab plus ipilimumab produced pathological responses in nearly all treated patients, including a high proportion of major pathological responses. These results demonstrate the biological sensitivity of localized dMMR tumors to immune-checkpoint blockade and support further evaluation of neoadjuvant immunotherapy, organ-preservation strategies, treatment duration, and the relationship between pathological response and long-term disease control.

NCT04969029 was registered as a randomized phase II study comparing adjuvant immunotherapy with standard chemotherapy after surgery for high-risk colon cancer characterized by MSI-H or *POLE/POLD1* alterations. The trial is relevant because it extends biomarker-directed immunotherapy into the adjuvant setting and includes ultramutated tumors beyond conventional MSI-H disease. However, the public registry record has not been updated since 2021 and currently lists the study status as unknown, with no posted results. It should therefore be described as a registered trial rather than as an actively recruiting or completed study unless a more recent authoritative update becomes available.

Anti-EGFR therapy may select resistant subclones harboring alterations in *RAS*, *BRAF*, the EGFR extracellular domain, *MET*, or *ERBB2*. Plasma-based studies have shown that selected resistance clones may emerge during treatment and decline after withdrawal, providing a biological rationale for molecularly selected anti-EGFR rechallenge [109,113].

### 7.5. Artificial Intelligence and Multi-Omics Implementation

Artificial intelligence and multi-omics approaches may integrate histopathological, radiological, genomic, transcriptomic, epigenetic, proteomic, microbiome, and clinical data into more comprehensive prediction models. However, high discriminatory performance in a retrospective development dataset is insufficient to establish clinical utility. Models should undergo independent external validation in populations that differ from the training cohort with respect to institution, geographical region, scanner or sequencing platform, specimen preparation, disease stage, demographic composition, and treatment pathway [114].

Dataset shift is a major implementation risk. An algorithm trained on high-quality surgical specimens from a specialist center may perform differently on small biopsies, older archived slides, locally advanced rectal tumors, or images produced by another scanner. Similarly, a model trained using one sequencing panel or RNA-processing pipeline may not remain calibrated when transferred to another laboratory. Performance should therefore be evaluated after deployment, and predefined procedures should be established for detecting calibration drift or degradation over time.

Bias may arise when relevant demographic, biological, or socioeconomic groups are under-represented in the development dataset. Aggregate accuracy can conceal clinically important differences in sensitivity, specificity, or calibration across sex, age, ancestry, tumor site, histological subtype, and healthcare setting. AI studies should report subgroup performance and should distinguish true biological variation from technical or access-related differences.

Transparency is also required regarding intended use, model inputs, training and validation datasets, reference standards, missing-data handling, uncertainty estimates, failure conditions, and the extent of human oversight. The level of explanation required depends on whether the system is used for research, triage, risk estimation, molecular-testing prioritization, or direct treatment recommendation. A model intended to support clinicians should not be presented as replacing pathological or molecular confirmation unless replacement has been prospectively validated.

Data protection and governance become particularly complex when genomic, imaging, and longitudinal clinical datasets are combined. De-identification, controlled access, audit trails, data-minimization principles, cybersecurity, and clearly defined responsibilities for secondary data use are required. Federated or distributed-learning approaches may reduce the need to centralize sensitive patient data, but they do not eliminate concerns regarding model leakage, site-specific bias, or governance.

The regulatory status of an AI system depends on its intended use and jurisdiction. Software that performs patient-specific analysis and provides information used for diagnosis or treatment may meet the definition of software as a medical device and may require risk-based regulatory review, quality-management procedures, lifecycle monitoring, and documentation of modifications. Regulatory guidance increasingly emphasizes transparency, representative data, assessment of bias, human-centered design, and monitoring throughout the total product lifecycle.

Until prospective clinical benefit has been demonstrated, AI-derived and multi-omics signatures should complement rather than replace validated pathological and molecular testing. Their most immediate role may be to prioritize confirmatory testing, identify complex interactions that cannot be captured by single biomarkers, and generate hypotheses for biomarker-stratified trials [115].

### 7.6. Measurable Research Priorities

Future research should move from broad aspirations toward prespecified and measurable implementation objectives.

First, assay harmonization should be evaluated using reproducible analytical indicators. Studies should report assay failure rates, limit of detection, minimum tumor fraction, interlaboratory concordance, repeat-test frequency, performance by specimen type, and the proportion of discordant results resolved by an orthogonal method. External quality assessment and reference materials should be incorporated whenever possible.

Second, clinical utility should be measured separately from analytical validity. Relevant endpoints include the proportion of patients whose treatment was changed by the biomarker result, the proportion receiving the recommended therapy, objective response, recurrence-free survival, overall survival, toxicity, quality of life, and avoidance of ineffective treatment. A biomarker should not be considered clinically implemented solely because it can be measured accurately.

Third, access should be monitored through pathway-level indicators. These include median turnaround time, proportion of results available before the treatment decision, assay-failure and repeat-biopsy rates, molecular tumor board referral, matched-trial referral, access to the recommended drug, and disparities according to institution, region, socioeconomic status, age, or other relevant patient characteristics.

Fourth, health-economic reporting should be standardized. Evaluations should include the direct assay cost, personnel and bioinformatic costs, confirmatory testing, counseling, downstream treatment, adverse-event management, avoided procedures, incremental cost per quality-adjusted life-year, and healthcare budget impact. Results should be reported for the specific healthcare system in which the strategy is intended to be implemented.

Fifth, biomarker-guided trials should use prospectively defined assays and decision algorithms. The biomarker threshold, sampling schedule, treatment assignment, management of indeterminate results, and primary clinical endpoint should be established before trial initiation. Central review, longitudinal biospecimen collection, and predefined analyses of treatment resistance should be incorporated where feasible.

Sixth, AI and multi-omics models should be evaluated beyond retrospective accuracy. Required outcomes include external multicenter performance, calibration, subgroup fairness, clinical workflow impact, rate of uninterpretable outputs, prospective treatment changes, post-deployment drift, and compliance with applicable regulatory and data-governance requirements.

Addressing these priorities would shift CRC precision oncology from biomarker accumulation toward evidence-based implementation. The most valuable future biomarkers will not necessarily be those that provide the most complex molecular description, but those that can be measured reproducibly, interpreted consistently, delivered in time, linked to an accessible intervention, and shown prospectively to improve outcomes that matter to patients [116,117].

**Table 4:** Representative Ongoing Biomarker-Guided Clinical Trials in Colorectal Cancer.

Trial and Identifier	Molecular/Clinical Population	Phase and Intervention	Principal Objective	Registry Status, July 2026
CheckMate 8HW—NCT04008030	MSI-H/dMMR unresectable or metastatic CRC	Phase III; nivolumab plus ipilimumab, nivolumab alone, or chemotherapy	Comparison of immunotherapy strategies with standard chemotherapy and long-term assessment of dual versus single-agent checkpoint inhibition	Active, not recruiting/closed to accrual
BREAKWATER—NCT04607421 [51]	Previously untreated BRAF V600E-mutated metastatic CRC	Phase III; encorafenib plus cetuximab with or without chemotherapy versus standard care	Evaluation of BRAF/EGFR-targeted treatment in the first-line setting	Active, not recruiting
CIRCULATE-US/NRG-GI008—NCT05174169	Resected high-risk stage II or stage III colon cancer, stratified by postoperative ctDNA	Phase II/III; ctDNA-guided treatment escalation or de-escalation	Determine whether postoperative molecular residual disease can guide the intensity of adjuvant chemotherapy	Recruiting
NICHE—NCT03026140	Early-stage colon cancer, including molecularly defined dMMR and pMMR cohorts	Phase II; neoadjuvant immune-checkpoint and novel immuno-oncology combinations	Evaluation of pathological response and molecular determinants of neoadjuvant immunotherapy sensitivity	Recruiting; selected cohorts remain open
Adjuvant immunotherapy versus chemotherapy—NCT04969029 [112]	Resected high-risk colon cancer with MSI-H or POLE/POLD1 alterations	Phase II; immunotherapy versus standard chemotherapy	Determine whether molecularly selected adjuvant immunotherapy improves outcomes	Unknown; registry record has not been recently verified
AZUR-1—NCT05723562 [83]	Untreated locally advanced dMMR/MSI-H rectal cancer	Phase II; dostarlimab monotherapy	Evaluate clinical complete response and the possibility of avoiding chemoradiotherapy and surgery	Active, not recruiting
AZUR-2—NCT05855200	Untreated T4N0 or stage III resectable dMMR/MSI-H colon cancer	Phase III; perioperative dostarlimab versus standard care	Determine whether perioperative immunotherapy improves event-free outcomes compared with surgery followed by chemotherapy or surveillance	Recruiting

Clinical-trial status is dynamic and should be reverified in the relevant registry immediately before final publication. “Active, not recruiting” indicates that enrollment has closed but trial-related treatment, follow-up, or outcome collection remains ongoing. “Unknown” indicates that the registry status has not been verified within the required reporting interval.

## 8. Integrated Discussion: Diagnostic, Prognostic, and Predictive Interpretation of Molecular Biomarkers

The clinical relevance of MSI/dMMR, RAS, *BRAF*, and HER2 should be interpreted according to their specific biomarker function. A diagnostic biomarker contributes to detecting or establishing the presence of disease, a prognostic biomarker provides information regarding the expected clinical course independently of a particular treatment, and a predictive biomarker identifies patients more or less likely to benefit from a specific therapeutic intervention. Although these categories may overlap, they should not be used interchangeably.

Most somatic alterations discussed in this review are detected after CRC has already been confirmed histopathologically. Therefore, *KRAS*, *NRAS*, *BRAF*, and HER2 alterations are not sufficiently sensitive or specific to serve as stand-alone diagnostic tests for CRC in asymptomatic individuals. These alterations occur only in molecular subsets of CRC and may also be identified in other tumor types. Their principal clinical value lies in molecular subclassification and therapeutic decision-making rather than primary cancer detection. Blood-based methylation, ctDNA, and multi-analyte assays may contribute to future non-invasive detection strategies, but these approaches are distinct from tumor-based mutation testing.

MSI/dMMR has the broadest multidimensional clinical role. In localized stage II colon cancer, MSI-H/dMMR is generally associated with a more favorable prognosis and with a lack of clinically meaningful benefit from fluoropyrimidine monotherapy. In metastatic disease, it is a strong predictive biomarker for immune-checkpoint inhibition. It also functions as a screening marker for Lynch syndrome, although tumor MSI-H or loss of an MMR protein does not independently confirm a germline syndrome. Clinical interpretation should integrate the pattern of MMR protein loss, *MLH1* promoter methylation, *BRAF* V600E status, family history, and germline testing when indicated.

*KRAS* and *NRAS* mutations are predominantly predictive rather than diagnostic biomarkers. Their most established function is to identify patients unlikely to benefit from anti-EGFR antibodies. Although several studies have investigated their association with survival, the independent prognostic effect of RAS mutations varies according to tumor stage, anatomical location, treatment exposure, and specific mutation. Accordingly, RAS status should primarily be presented as an exclusionary treatment-selection biomarker rather than a universally adverse prognostic factor [28].

*BRAF* V600E has both prognostic and predictive relevance. It is associated with a biologically distinct and frequently aggressive CRC subtype, particularly in microsatellite-stable metastatic disease. At the same time, it identifies patients eligible for combined *BRAF* and EGFR inhibition. Its interpretation is influenced by MSI/MMR status because *BRAF* V600E frequently coexists with sporadic *MLH1* promoter methylation. In a tumor with loss of *MLH1/PMS2* expression, detection of *BRAF* V600E supports a sporadic pathway, although it does not by itself exclude every possibility of hereditary predisposition. The treatment-predictive role of *BRAF* V600E has been validated through BEACON CRC and BREAKWATER.

HER2 amplification is principally a predictive biomarker. It identifies a small subgroup of patients, most often within *RAS/BRAF* wild-type disease, who may benefit from HER2-directed therapy. HER2 activation may also provide a bypass signaling mechanism contributing to primary or acquired resistance to EGFR inhibition. However, the independent prognostic effect of HER2 amplification remains less consistent than that of *BRAF* V600E, and HER2 status should not be presented as a validated population-level diagnostic or universally prognostic marker. Its strongest current clinical role is selection for molecularly matched therapy, as supported by HERACLES and MOUNTAINEER [22].

These alterations should also be interpreted in relation to one another. *KRAS* and *NRAS* mutations activate signaling upstream of RAF, whereas *BRAF* V600E activates the pathway downstream of RAS. These driver alterations are usually, although not invariably, found in separate molecular subgroups. HER2 amplification provides receptor-level activation of both MAPK and PI3K–AKT signaling and is clinically most relevant after confirmation of *RAS/BRAF* wild-type status. MSI/dMMR represents a separate DNA-repair phenotype but may overlap with *BRAF* V600E in sporadic *MLH1*-methylated tumors. Consequently, a sequential or panel-based molecular algorithm is more informative than interpretation of any marker in isolation [15].

A clinically oriented testing pathway should first establish MSI/MMR status because of its implications for Lynch syndrome evaluation, prognosis in localized disease, and immunotherapy eligibility. In metastatic disease, extended RAS and BRAF testing should be performed to guide anti-EGFR and *BRAF*-targeted strategies. HER2 testing should be considered particularly in *RAS/BRAF* wild-type tumors in which HER2-directed treatment may become relevant. Longitudinal ctDNA analysis may subsequently identify clonal evolution and acquired resistance, but it should complement rather than universally replace tissue-based molecular testing.

Overall, these biomarkers are most clinically valuable when their precise function is clearly specified. MSI/dMMR may be prognostic, predictive, and relevant to hereditary-risk screening; RAS mutations are principally negative predictive biomarkers; *BRAF* V600E is both adverse prognostic and therapeutically predictive; and HER2 amplification is primarily predictive for targeted therapy. None of these alterations should be used alone as a population-level diagnostic test for CRC.

### Limitations of the Review

This review has several limitations. It was designed as a structured narrative review rather than a systematic review or meta-analysis. The literature-identification strategy was not prospectively registered, source selection and data interpretation were not performed through formal duplicate independent screening, and no standardized risk-of-bias or certainty-of-evidence assessment was undertaken. Consequently, selection and interpretation bias cannot be excluded. The thematic emphasis of the review should not be interpreted as a quantitative estimate of the volume or comparative strength of evidence available for individual biomarkers. In addition, the proposed clinical implementation framework is intended as a practical organizational tool and not as a substitute for guideline recommendations or validated alteration-level systems such as ESCAT and OncoKB. Finally, because molecular diagnostics, regulatory approvals, and biomarker-guided trials are evolving rapidly, some therapeutic and implementation statements may require periodic updating.

Despite these measures, the purposive nature of source selection may have favored more clinically influential or recently published studies. The review should therefore be interpreted as a structured and clinically oriented narrative synthesis rather than an exhaustive quantitative assessment of all available publications.

## 9. Conclusions

The molecular characterization of CRC has fundamentally transformed disease classification and therapeutic decision-making. Biomarkers such as MSI/dMMR, *KRAS*, *NRAS*, *BRAF*, HER2, and *NTRK* have become integral components of routine clinical practice, enabling more accurate prognostic assessment and personalized treatment selection. The success of immune checkpoint inhibitors in MSI-H/dMMR tumors and the development of targeted therapies for specific molecular subgroups illustrate the growing impact of precision oncology in CRC management.

At the same time, emerging biomarkers including tumor mutational burden, *POLE/POLD1* mutations, circulating tumor DNA, DNA damage repair alterations, transcriptomic signatures, microbiome-derived biomarkers, epigenetic markers, and artificial intelligence-based prediction models are expanding the boundaries of molecular stratification. Although many of these approaches remain investigational, they offer promising opportunities to refine patient selection, improve treatment monitoring, and identify novel therapeutic targets.

The future of CRC management will likely depend not on individual biomarkers, but on the integration of genomic, epigenetic, transcriptomic, immune, microbiome, radiomic, and liquid biopsy-derived data into unified precision oncology platforms. Such multidimensional approaches have the potential to capture tumor complexity more accurately than any single molecular alteration alone.

As molecular technologies continue to evolve, precision oncology is expected to transition from static biomarker assessment toward dynamic, longitudinal disease monitoring supported by artificial intelligence and multi-omics integration. Ultimately, these advances may facilitate truly individualized treatment strategies and contribute to improved outcomes for patients with CRC.

## Figures and Tables

**Table 3:** Selected Emerging Biomarkers in CRC: Potential Clinical Applications and Current Implementation Barriers.

Biomarker or Molecular Approach	Current Evidence Status	Potential Clinical Application	Principal Implementation Barriers	Representative Evidence
Circulating tumor DNA (ctDNA)	Advanced clinical validation; strong prognostic value for postoperative molecular residual disease, with treatment-guiding utility under prospective evaluation	Detection of molecular residual disease after curative-intent surgery; postoperative recurrence-risk stratification; adjuvant-treatment escalation or de-escalation; longitudinal monitoring of response and acquired resistance	Differences between tumor-informed and tumor-agnostic assays; variable tumor shedding; timing of blood collection; false-negative results in low-volume or low-shedding disease; absence of universally accepted positivity thresholds; uncertainty regarding the optimal intervention for persistent or newly positive ctDNA	The randomized DYNAMIC trial showed that ctDNA-guided management reduced adjuvant chemotherapy use in stage II colon cancer without compromising recurrence-free survival; CIRCULATE-US/NRG-GI008 is prospectively evaluating ctDNA-guided escalation and de-escalation.
Tumor mutational burden (TMB)	Context-dependent biomarker; a tumor-agnostic regulatory precedent exists, but CRC-specific predictive utility remains insufficiently validated, particularly in microsatellite-stable disease	Refinement of immunotherapy selection in MSI-H/dMMR tumors and identification of selected hypermutated MSS tumors that may exhibit increased immunogenicity	Assay- and panel-dependent estimates; inconsistent cutoffs; incomplete harmonization between tissue and plasma TMB; biological dependence on the underlying mutational process; overlap with MSI and POLE/POLD1 alterations; limited efficacy of checkpoint inhibition in many MSS/TMB-high CRCs	Pembrolizumab has a tumor-agnostic TMB-high indication in selected jurisdictions, but recent CRC-specific evidence indicates that MSS/TMB-high status alone may not reliably identify patients who benefit from pembrolizumab.
Pathogenic POLE/POLD1 proofreading-domain alterations	Rare but clinically promising predictive biomarkers; evidence is strongest for pathogenic exonuclease-domain variants producing a proofreading-deficient ultramutated phenotype	Identification of selected MSS ultramutated tumors with potential sensitivity to immune-checkpoint inhibitors; refinement of hereditary-risk assessment in selected patients	Very low prevalence; difficulty distinguishing pathogenic proofreading defects from passenger variants; frequent variants of uncertain significance; lack of standardized functional classification; limited prospective trial evidence	A global cohort of patients with proofreading-deficient POLE/POLD1-mutated metastatic CRC demonstrated favorable responses and survival with immune-checkpoint inhibitors; prospective evaluation remains limited.
DNA damage repair and homologous recombination deficiency signatures	Exploratory; biological and preclinical rationale exists, but no validated CRC-specific treatment-selection role has been established	Identification of tumors potentially susceptible to synthetic-lethality strategies, including PARP inhibition; possible refinement of immunotherapy or combination-treatment selection	Heterogeneity of the genes included in DDR panels; uncertain functional significance of individual variants; lack of standardized HRD thresholds in CRC; limited evidence that genomic DDR alterations consistently predict treatment benefit; absence of established CRC-specific therapeutic algorithms	Experimental CRC studies have identified homologous-recombination-deficient subgroups potentially sensitive to PARP inhibition, but prospective clinical validation is required.
Consensus Molecular Subtypes and other transcriptomic signatures	Biologically validated classification systems with demonstrated prognostic associations; not routinely implemented as treatment-selection assays	Functional tumor classification; prognostic stratification; identification of immune, mesenchymal, metabolic, and canonical biological phenotypes; generation of hypotheses for treatment selection	Dependence on RNA quality and tissue processing; intratumoral heterogeneity; stromal contamination; differences between primary and metastatic specimens; classifier discordance; lack of prospective evidence that CMS-guided treatment improves outcomes	CMS classifiers have been evaluated in large clinico-genomic datasets, but their predictive utility and capacity to direct routine treatment remain incompletely established.
Spatial transcriptomic and immune-microenvironment signatures	Early translational research	Characterization of spatial tumor–immune and tumor–stromal interactions; identification of resistance niches; refinement of immunotherapy-response prediction; discovery of new therapeutic targets	High cost; complex tissue preparation; restricted availability; spatial sampling bias; lack of standardized analytical pipelines; computational burden; limited reproducibility and absence of validated clinical thresholds	Spatial and single-cell analyses have identified distinct immune and stromal niches within CRC, but current applications remain primarily biological and hypothesis-generating.
Epigenetic and circulating methylation signatures	Selected methylation assays have clinical or regulatory precedent for CRC detection, whereas broader methylome signatures remain investigational	Non-invasive CRC detection; risk stratification; characterization of CIMP-related biology; potential postoperative surveillance and recurrence assessment	Variable sensitivity for precursor and early-stage lesions; biological and technical heterogeneity; bisulfite-conversion and assay-standardization requirements; uncertain incremental benefit over established screening methods; limited evidence for treatment selection	Plasma methylated SEPT9 has undergone prospective evaluation for CRC screening, while broader circulating methylation signatures require additional clinical validation.
Microbiome-derived signatures	Early translational and observational evidence; no standardized clinical biomarker is currently established	Non-invasive CRC risk assessment or detection; prognostic stratification; characterization of host–tumor interactions; possible prediction of treatment toxicity or immunotherapy response	Major effects of diet, medication, antibiotics, geography, age, sampling, storage, sequencing platform, and bioinformatic pipeline; compositional rather than absolute abundance data; limited causal evidence; poor inter-cohort reproducibility	Reproducible associations have been reported for selected microbial taxa, but large studies also demonstrate substantial confounding and methodological variability that currently limit clinical implementation.
Artificial intelligence-based digital pathology biomarkers	Retrospectively and externally validated for selected tasks, particularly MSI prescreening; not yet a replacement for molecular testing	Prescreening or prioritization for MSI/dMMR testing; molecular-subtype prediction from routine H&E slides; prognosis estimation; treatment-response modeling	Dataset shift between institutions; differences in staining, scanners, tissue processing, and patient populations; algorithmic bias; limited explainability; uncertain failure conditions; regulatory requirements; need for prospective workflow and outcome validation	Multicenter and external-validation studies support AI-based MSI prescreening, but confirmatory IHC, PCR, or NGS remains necessary for clinical treatment decisions.
Radiomics and radiogenomics	Exploratory; predominantly based on retrospective model-development and validation studies	Non-invasive prediction of MSI status and other molecular phenotypes; preoperative risk stratification; assessment of treatment response and tumor heterogeneity	-	-

Abbreviations: AI, artificial intelligence; CIMP, CpG island methylator phenotype; CMS, Consensus Molecular Subtypes; CRC, colorectal cancer; ctDNA, circulating tumor DNA; DDR, DNA damage repair; dMMR, deficient mismatch repair; H&E, hematoxylin and eosin; HRD, homologous recombination deficiency; MSI-H, microsatellite instability-high; MSS, microsatellite stable; NGS, next-generation sequencing; PCR, polymerase chain reaction; TMB, tumor mutational burden. The evidence-status descriptions are intended to indicate the current degree of clinical development and should not be interpreted as formal evidence levels. Inclusion in this table does not imply guideline endorsement or routine clinical use. Several biomarker categories overlap biologically, and individual tumors may simultaneously exhibit more than one molecular feature.

## Data Availability

No new data were created or analyzed in this study. Data sharing is not applicable to this article.

## Abbreviations

The following abbreviations are used in this manuscript:

ADC	Antibody–Drug Conjugate
AI	Artificial Intelligence
AKT	Protein Kinase B
BRAF	v-Raf Murine Sarcoma Viral Oncogene Homolog B1
CIMP	CpG Island Methylator Phenotype
CIN	Chromosomal Instability
CMS	Consensus Molecular Subtype
CRC	Colorectal Cancer
ctDNA	Circulating Tumor DNA
DDR	DNA Damage Repair
dMMR	Deficient Mismatch Repair
DNA	Deoxyribonucleic Acid
EGFR	Epidermal Growth Factor Receptor
ERBB2	Erb-B2 Receptor Tyrosine Kinase 2
FISH	Fluorescence In Situ Hybridization
HER2	Human Epidermal Growth Factor Receptor 2
ICI	Immune Checkpoint Inhibitor
IHC	Immunohistochemistry
ISH	In Situ Hybridization
KRAS	Kirsten Rat Sarcoma Viral Oncogene Homolog
lncRNA	Long Non-Coding RNA
MAPK	Mitogen-Activated Protein Kinase
miRNA	MicroRNA
MLH1	MutL Homolog 1
mCRC	Metastatic Colorectal Cancer
MRD	Minimal Residual Disease
MSH2	MutS Homolog 2
MSH6	MutS Homolog 6
MSI	Microsatellite Instability
MSI-H	Microsatellite Instability-High
MSS	Microsatellite Stable
ncRNA	Non-Coding RNA
NGS	Next-Generation Sequencing
NRAS	Neuroblastoma Rat Sarcoma Viral Oncogene Homolog
NTRK	Neurotrophic Tyrosine Receptor Kinase
PARP	Poly(ADP-Ribose) Polymerase
PCR	Polymerase Chain Reaction
PD-1	Programmed Cell Death Protein 1
PD-L1	Programmed Death-Ligand 1
PI3K	Phosphoinositide 3-Kinase
PMS2	PMS1 Homolog 2, Mismatch Repair System Component
POLE	DNA Polymerase Epsilon Catalytic Subunit
POLD1	DNA Polymerase Delta 1 Catalytic Subunit
RNA	Ribonucleic Acid
TMB	Tumor Mutational Burden
TRK	Tropomyosin Receptor Kinase

## References

[1] Rodríguez M.R., Biedma B.A., Rodríguez Pérez I., Romeo J.A. (2025). Elucidating the role of kras, nras, and braf mutations and microsatellite instability in colorectal cancer via next-generation sequencing. Cancers.

[2] Kojitani Y., Takeda M. (2025). Current status of precision medicine in colorectal cancer in japan. Int. J. Mol. Sci..

[3] Patel A., Gulhati P. (2024). Molecular landscape and therapeutic strategies against colorectal cancer. Cancers.

[4] Sung H., Filho A.M., Laversanne M., Ferlay J., Siegel R.L., Soerjomataram I., Jemal A., Bray F. (2026). Global Cancer Statistics 2024: Globocan estimates of incidence and mortality worldwide for 34 cancers in 186 countries. CA Cancer J. Clin..

[5] Puccini A., Seeber A., Berger M.D. (2022). Biomarkers in metastatic colorectal cancer: Status quo and future perspective. Cancers.

[6] Mateo J., Chakravarty D., Dienstmann R., Jezdic S., Gonzalez-Perez A., Lopez-Bigas N., Ng C.K.Y., Bedard P.L., Tortora G., Douillard J.Y. (2018). A framework to rank genomic alterations as targets for cancer precision medicine: The esmo scale for clinical actionability of molecular targets (escat). Ann. Oncol..

[7] Chakravarty D., Gao J., Phillips S.M., Kundra R., Zhang H., Wang J., Rudolph J.E., Yaeger R., Soumerai T., Nissan M.H. (2017). Oncokb: A precision oncology knowledge base. JCO Precis. Oncol..

[8] Salva de Torres C., Baraibar I., González N.S., Ros J., Salva F., Rodríguez-Castells M., Alcaraz A., García A., Tabernero J., Élez E. (2024). Current and emerging treatment paradigms in colorectal cancer: Integrating hallmarks of cancer. Int. J. Mol. Sci..

[9] Kong L., Yiu C.H., Lu C.Y. (2025). Effectiveness and safety of immune checkpoint inhibitors in colorectal cancer: A systematic review of real-world studies. Curr. Oncol. Rep..

[10] Lebedeva A., Taraskina A., Grigoreva T., Belova E., Kuznetsova O., Ivanilova D., Sergeeva A., Kavun A., Veselovsky E., Nikulin V. (2025). The role of msi testing methodology and its heterogeneity in predicting colorectal cancer immunotherapy response. Int. J. Mol. Sci..

[11] Grau Béjar J.F., Galende E.Y., Genestie C., Blanc-Durand F., Le Formal A., Rouleau É., Leary A. (2026). Predictive biomarkers of response to immune checkpoint inhibitors in mismatch repair-deficient endometrial cancer. Ther. Adv. Med. Oncol..

[12] Amato M., Franco R., Facchini G., Addeo R., Ciardiello F., Berretta M., Vita G., Sgambato A., Pignata S., Caraglia M. (2022). Microsatellite instability: From the implementation of the detection to a prognostic and predictive role in cancers. Int. J. Mol. Sci..

[13] Xu B., Lian J., Pang X., Gu Y., Zhu J., Zhang Y., Lu H. (2024). Identification of colon cancer subtypes based on multi-omics data—Construction of methylation markers for immunotherapy. Front. Oncol..

[14] Kim J.H., Hong J., Lee J.A., Jung M., Choi E., Cho N.Y., Kang G.H., Kim S. (2024). Immune microenvironmental heterogeneity according to tumor DNA methylation phenotypes in microsatellite instability-high colorectal cancers. Cancer Immunol. Immunother..

[15] De Roock W., Claes B., Bernasconi D., De Schutter J., Biesmans B., Fountzilas G., Kalogeras K.T., Kotoula V., Papamichael D., Laurent-Puig P. (2010). Effects of kras, braf, nras, and pik3ca mutations on the efficacy of cetuximab plus chemotherapy in chemotherapy-refractory metastatic colorectal cancer: A retrospective consortium analysis. Lancet Oncol..

[16] Fakih M., Salvatore L., Esaki T., Modest D.P., Lopez-Bravo D.P., Taieb J., Karamouzis M., Ruiz-Garcia E., Kim T.W., Kuboki Y. (2024). Overall survival (os) of phase 3 codebreak 300 study of sotorasib plus panitumumab (soto+ pani) versus investigator’s choice of therapy for kras g12c-mutated metastatic colorectal cancer (mcrc). Am. Soc. Clin. Oncol..

[17] Lastraioli E., Bettiol A., Iorio J., Limatola E., Checcacci D., Parisi E., Bianchi C., Arcangeli A., Iannopollo M., Di Costanzo F. (2023). Evaluation of ras mutational status in liquid biopsy to monitor disease progression in metastatic colorectal cancer patients. Cells.

[18] Napolitano S., Woods M., Lee H.M., De Falco V., Martini G., Della Corte C.M., Martinelli E., Famiglietti V., Ciardiello D., Anderson A. (2023). Antitumor efficacy of dual blockade with encorafenib+ cetuximab in combination with chemotherapy in human braf v600e-mutant colorectal cancer. Clin. Cancer Res..

[19] Yoshino T., Di Bartolomeo M., Raghav K., Masuishi T., Loupakis F., Kawakami H., Yamaguchi K., Nishina T., Wainberg Z., Elez E. (2023). Final results of destiny-crc01 investigating trastuzumab deruxtecan in patients with her2-expressing metastatic colorectal cancer. Nat. Commun..

[20] Ratti M., Grizzi G., Passalacqua R., Lampis A., Cereatti F., Grassia R., Hahne J.C. (2021). NTRK fusions in colorectal cancer: Clinical meaning and future perspective. Expert. Opin. Ther. Targets..

[21] Marabelle A., Fakih M., Lopez J., Shah M., Shapira-Frommer R., Nakagawa K., Chung H.C., Kindler H.L., Lopez-Martin J.A., Miller W.H. (2020). Association of tumour mutational burden with outcomes in patients with advanced solid tumours treated with pembrolizumab: Prospective biomarker analysis of the multicohort, open-label, phase 2 keynote-158 study. Lancet Oncol..

[22] Sartore-Bianchi A., Trusolino L., Martino C., Bencardino K., Lonardi S., Bergamo F., Zagonel V., Leone F., Depetris I., Martinelli E. (2016). Dual-targeted therapy with trastuzumab and lapatinib in treatment-refractory, kras codon 12/13 wild-type, her2-positive metastatic colorectal cancer (heracles): A proof-of-concept, multicentre, open-label, phase 2 trial. Lancet Oncol..

[23] Leader A.M., Grout J.A., Maier B.B., Nabet B.Y., Park M.D., Tabachnikova A., Chang C., Walker L., Lansky A., Le Berichel J. (2021). Single-cell analysis of human non-small cell lung cancer lesions refines tumor classification and patient stratification. Cancer Cell.

[24] Gustav M., Reitsam N.G., Carrero Z.I., Loeffler C.M., van Treeck M., Yuan T., West N.P., Quirke P., Brinker T.J., Brenner H. (2024). Deep learning for dual detection of microsatellite instability and pole mutations in colorectal cancer histopathology. npj Precis. Oncol..

[25] LaPelusa M., Qiao W., Iorgulescu B., Lucas F.S., Patel K., Bhamidipati D., Thomas J.V., You N., Foo W.C., Maru D. (2025). Long-term efficacy of pembrolizumab and the clinical utility of ctdna in locally advanced dmmr/msi-h solid tumors. Nat. Commun..

[26] He R., Zhang H., Zhao H., Yin X., Lu J., Gu C., Gao J., Xu Q. (2023). Multiomics analysis reveals cuproptosis-related signature for evaluating prognosis and immunotherapy efficacy in colorectal cancer. Cancers.

[27] Kim J.W., Lee H.J., Lee J.Y., Park S.R., Kim Y.J., Hwang I.G., Bae W.K., Byun J.H., Kim J.S., Kang E.J. (2024). Phase ii study of nivolumab in patients with genetic alterations in DNA damage repair and response who progressed after standard treatment for metastatic solid cancers (km-06). J. Immunother. Cancer.

[28] Guinney J., Dienstmann R., Wang X., de Reyniès A., Schlicker A., Soneson C., Marisa L., Roepman P., Nyamundanda G., Angelino P. (2015). The consensus molecular subtypes of colorectal cancer. Nat. Med..

[29] Ribic Christine M., Daniel J.S., Malcolm J.M., Stephen N.T., Amy J.F., Richard M.G., Stanley R.H., Laurent-Puig P., Gryfe R., Lois E.S. (2003). Tumor microsatellite-instability status as a predictor of benefit from fluorouracil-based adjuvant chemotherapy for colon cancer. N. Engl. J. Med..

[30] Rat L.A., Moldovan A.F., Trifan D.F., Matiș L., Murvai G.F., Maris L., Ghitea T.C., Maghiar M.A. (2023). Can the correlation of periodontopathies with gastrointestinal diseases be used as indicators in severe colorectal diseases?. Biomedicines.

[31] Song L., Yu H., Jia J., Li Y. (2017). A systematic review of the performance of the sept9 gene methylation assay in colorectal cancer screening, monitoring, diagnosis and prognosis. Cancer Biomark..

[32] Schee K., Fodstad Ø., Flatmark K. (2010). MicroRNAs as Biomarkers in Colorectal Cancer. Am. J. Pathol..

[33] Roggia C., Armeanu-Ebinger S., Gschwind A., Seibel-Kelemen O., Hertler S., Faust U., Liebmann A., Haack T.B., Neumann M., Bonzheim I. (2023). Germline findings in patients with advanced malignancies screened with paired blood-tumour testing for personalised treatment approaches. Eur. J. Cancer.

[34] Diwan H., Mehta A., Sharma S., Mattoo S., Agnihotri S. (2026). Integrated tumor and germline profiling of lynch syndrome in a north indian cohort. Front. Oncol..

[35] Monahan K.J., Bradshaw N., Dolwani S., Desouza B., Dunlop M.G., East J.E., Ilyas M., Kaur A., Lalloo F., Latchford A. (2020). Guidelines for the management of hereditary colorectal cancer from the british society of gastroenterology (bsg)/association of coloproctology of great britain and ireland (acpgbi)/united kingdom cancer genetics group (ukcgg). Gut.

[36] Tabernero J., Velez L., Trevino T., Grothey A., Yaeger R., Van Cutsem E., Wasan H., Desai J., Ciardiello F., Yoshino T. (2021). Management of adverse events from the treatment of encorafenib plus cetuximab for patients with braf v600e-mutant metastatic colorectal cancer: Insights from the beacon crc study. ESMO Open.

[37] Pokorná M., Černá M., Boussios S., Ovsepian S.V., O’Leary V.B. (2024). Lncrna biomarkers of glioblastoma multiforme. Biomedicines.

[38] Saillard C., Dubois R., Tchita O., Loiseau N., Garcia T., Adriansen A., Carpentier S., Reyre J., Enea D., von Loga K. (2023). Validation of msintuit as an ai-based pre-screening tool for msi detection from colorectal cancer histology slides. Nat. Commun..

[39] Taieb J., Ambrosini M., Alouani E., Lonardi S., Sinicrope F.A., Decraecker M., Boileve A., Hafliger E., Mazard T., Pernot S. (2025). Early treatment discontinuation in patients with deficient mismatch repair or microsatellite instability high metastatic colorectal cancer receiving immune checkpoint inhibitors. J. Immunother. Cancer.

[40] Brahmer J.R., Lee J.S., Ciuleanu T.E., Bernabe Caro R., Nishio M., Urban L., Audigier-Valette C., Lupinacci L., Sangha R., Pluzanski A. (2023). Five-Year Survival Outcomes with Nivolumab Plus Ipilimumab Versus Chemotherapy as First-Line Treatment for Metastatic Non-Small-Cell Lung Cancer in CheckMate 227. J. Clin. Oncol..

[41] Strickler J.H., Bekaii-Saab T., Cercek A., Heinemann V., Nakamura Y., Raghav K., Siena S., Tabernero J., Van Cutsem E., Yoshino T. (2025). Mountaineer-03 phase iii study design: First-line mfolfox6 + tucatinib + trastuzumab for her2+ metastatic colorectal cancer. Future Oncol..

[42] Loukovaara M., Pasanen A., Bützow R. (2021). Mismatch repair deficiency as a predictive and prognostic biomarker in molecularly classified endometrial carcinoma. Cancers.

[43] Fountzilas E., Papadopoulos T., Papadopoulou E., Gouedard C., Kourea H.P., Constantoulakis P., Magkou C., Sfakianaki M., Kotoula V., Bantouna D. (2024). Nationwide real-world data of microsatellite instability and/or mismatch repair deficiency in cancer: Prevalence and testing patterns. Diagnostics.

[44] Cercek A., Foote M.B., Rousseau B., Smith J.J., Shia J., Sinopoli J., Weiss J., Lumish M., Temple L., Patel M. (2025). Nonoperative Management of Mismatch Repair-Deficient Tumors. N. Engl. J. Med..

[45] Zwart K., van der Baan F.H., Punt C.J.A., Wensink G.E., Bolhuis K., Laclé M.M., van Grevenstein W.M.U., Hagendoorn J., de Hingh I.H., Koopman M. (2023). Survival of patients with deficient mismatch repair versus proficient mismatch repair metastatic colorectal cancer receiving curative-intent local treatment of metastases in a nationwide cohort. Ann. Surg. Oncol..

[46] André T., Shiu K.K., Kim T.W., Jensen B.V., Jensen L.H., Punt C., Smith D., Garcia-Carbonero R., Benavides M., Gibbs P. (2020). Pembrolizumab in microsatellite-instability-high advanced colorectal cancer. N. Engl. J. Med..

[47] Schrock A.B., Ouyang C., Sandhu J., Sokol E., Jin D., Ross J.S., Miller V.A., Lim D., Amanam I., Chao J. (2019). Tumor mutational burden is predictive of response to immune checkpoint inhibitors in msi-high metastatic colorectal cancer. Ann. Oncol..

[48] Van Cutsem E., Eng C., Nowara E., Świeboda-Sadlej A., Tebbutt N.C., Mitchell E., Davidenko I., Stephenson J., Elez E., Prenen H. (2014). Randomized phase ib/ii trial of rilotumumab or ganitumab with panitumumab versus panitumumab alone in patients with wild-type kras metastatic colorectal cancer. Clin. Cancer Res..

[49] Desai J., Alonso G., Kim S.H., Cervantes A., Karasic T., Medina L., Shacham-Shmueli E., Cosman R., Falcon A., Gort E. (2024). Divarasib plus cetuximab in kras g12c-positive colorectal cancer: A phase 1b trial. Nat. Med..

[50] Kopetz S., Grothey A., Yaeger R., Van Cutsem E., Desai J., Yoshino T., Wasan H., Ciardiello F., Loupakis F., Yong S.H. (2019). Encorafenib, binimetinib, and cetuximab in braf v600e–mutated colorectal cancer. N. Engl. J. Med..

[51] Rimbert J., Tachon G., Junca A., Villalva C., Karayan-Tapon L., Tougeron D. (2018). Association between clinicopathological characteristics and ras mutation in colorectal cancer. Mod. Pathol..

[52] Osumi H., Takashima A., Ooki A., Yoshinari Y., Wakatsuki T., Hirano H., Nakayama I., Okita N., Sawada R., Ouchi K. (2023). A multi-institutional observational study evaluating the incidence and the clinicopathological characteristics of neoras wild-type metastatic colorectal cancer. Transl. Oncol..

[53] Zhong J., Sun Z., Li S., Yang L., Cao Y., Bao J. (2023). Immune checkpoint blockade therapy for braf mutant metastatic colorectal cancer: The efficacy, new strategies, and potential biomarkers. Discov. Oncol..

[54] Colombo A., Concetta P.M., Gebbia V., Sambataro D., Scandurra G., Valerio M.R. (2025). A narrative review of the role of immunotherapy in metastatic carcinoma of the colon harboring a braf mutation. In Vivo.

[55] Char S.K., Singh H., Ng K. (2026). Biomarkers for early detection and monitoring of colorectal cancer. Gastroenterol. Clin..

[56] Chen F.-L., Wang Y.-Y., Liu W., Xing B.-C. (2022). Prognostic factors in colorectal liver metastases patients with various tumor numbers treated by liver resection: A single-center, retrospective study. World J. Surg. Oncol..

[57] Bonilla C.E., Montenegro P., O’Connor J.M., Hernando-Requejo O., Aranda E., Llerena J.P., Llontop A., Escobar J.G., Romero M.D.C.D., Hernandez Y.B. (2023). Ibero-american consensus review and incorporation of new biomarkers for clinical practice in colorectal cancer. Cancers.

[58] Jourdain H., Di Meglio A., Mansouri I., Desplas D., Zureik M., Haddy N. (2025). Real-world efficacy and safety of trastuzumab deruxtecan versus trastuzumab emtansine and tucatinib as second-line and third-line treatments for her2-positive metastatic breast cancer: Two target trial emulation studies. Lancet Reg. Health–Eur..

[59] Kivrak H., Ozakinci H., Karasoy D., Sak S.D. (2022). Her2 amplification by next-generation sequencing in lung carcinoma: A comparison of ngs amplified and non-amplified cases by immunohistochemistry and in situ hybridization. Balk. Med. J..

[60] Kawczak P., Bączek T. (2026). Molecular targeting of egfr, braf, and her2 signaling in colorectal cancer: Contemporary advances with panitumumab, encorafenib, and tucatinib. J. Clin. Med..

[61] Bebb D.G., Banerji S., Blais N., Desmeules P., Gill S., Grin A., Feilotter H., Hansen A.R., Hyrcza M., Krzyzanowska M. (2021). Canadian consensus for biomarker testing and treatment of trk fusion cancer in adults. Curr. Oncol..

[62] Drilon A. (2019). Trk inhibitors in trk fusion-positive cancers. Ann. Oncol..

[63] Harada G., Drilon A. (2022). Trk inhibitor activity and resistance in trk fusion-positive cancers in adults. Cancer Genet..

[64] Yan H., Song L., Li Y., Xu Q., Guo W., Lin S., Jiang W., Wang Z., Deng L., Huang Z. (2024). Clinical evidence for efficacy of pembrolizumab in msi-h and tmb-h advanced solid tumor: Results from three cancer centers in china. Cancer Immunol. Immunother..

[65] Sakakida T., Ishikawa T., Doi T., Morita R., Kataoka S., Miyake H., Yamaguchi K., Moriguchi M., Sogame Y., Yasuda H. (2024). Genomic profile and clinical features of msi-h and tmb-high pancreatic cancers: Real-world data from c-cat database. J. Gastroenterol..

[66] Woo H.G., Park E.S., Thorgeirsson S.S., Kim Y.J. (2011). Exploring genomic profiles of hepatocellular carcinoma. Mol. Carcinog..

[67] Ullah I., Yang L., Yin F.-T., Sun Y., Li X.-H., Li J., Wang X.-J. (2022). Multi-omics approaches in colorectal cancer screening and diagnosis, recent updates and future perspectives. Cancers.

[68] Ye P., Cai P., Xie J., Wei Y. (2021). The diagnostic accuracy of digital pcr, arms and ngs for detecting kras mutation in cell-free DNA of patients with colorectal cancer: A systematic review and meta-analysis. PLoS ONE.

[69] Valladares-Ayerbes M., Safont M.J., Flores E.G., García-Alfonso P., Aranda E., Muñoz A.-M.L., Ferrer E.F., Nogueras L.C., Rodríguez-Salas N., Aparicio J. (2024). Sequential ras mutations evaluation in cell-free DNA of patients with tissue ras wild-type metastatic colorectal cancer: The perseida (cohort 2) study. Clin. Transl. Oncol..

[70] Xiao J., Li W., Huang Y., Huang M., Li S., Zhai X., Zhao J., Gao C., Xie W., Qin H. (2021). A next-generation sequencing-based strategy combining microsatellite instability and tumor mutation burden for comprehensive molecular diagnosis of advanced colorectal cancer. BMC Cancer.

[71] Pesola G., Epistolio S., Cefalì M., Trevisi E., De Dosso S., Frattini M. (2024). Neo-ras wild type or ras conversion in metastatic colorectal cancer: A comprehensive narrative review. Cancers.

[72] Diaz L.A., Williams R.T., Wu J., Kinde I., Hecht J.R., Berlin J., Allen B., Bozic I., Reiter J.G., Nowak M.A. (2012). The molecular evolution of acquired resistance to targeted egfr blockade in colorectal cancers. Nature.

[73] Awwad S.W., Serrano-Benitez A., Thomas J.C., Gupta V., Jackson S.P. (2023). Revolutionizing DNA repair research and cancer therapy with crispr–cas screens. Nat. Rev. Mol. Cell Biol..

[74] Pavelescu L.A., Enache R.M., Roşu O.A., Profir M., Creţoiu S.M., Gaspar B.S. (2024). Predictive biomarkers and resistance mechanisms of checkpoint inhibitors in malignant solid tumors. Int. J. Mol. Sci..

[75] Uhlik M., Pointing D., Iyer S., Ausec L., Štajdohar M., Cvitkovič R., Žganec M., Culm K., Santos V.C., Pytowski B. (2023). Xerna™ tme panel is a machine learning-based transcriptomic biomarker designed to predict therapeutic response in multiple cancers. Front. Oncol..

[76] Xu Q., Xu H., Deng R., Wang Z., Li N., Qi Z., Zhao J., Huang W. (2021). Multi-omics analysis reveals prognostic value of tumor mutation burden in hepatocellular carcinoma. Cancer Cell Int..

[77] Heregger R., Huemer F., Steiner M., Gonzalez-Martinez A., Greil R., Weiss L. (2023). Unraveling resistance to immunotherapy in msi-high colorectal cancer. Cancers.

[78] Hildebrand L.A., Pierce C.J., Dennis M., Paracha M., Maoz A. (2021). Artificial intelligence for histology-based detection of microsatellite instability and prediction of response to immunotherapy in colorectal cancer. Cancers.

[79] Zhang Y., Zhang X., Zhong X., Huang L., Jiang W., Zhang C., Liu L., You R., Li Y., Yi X. (2025). Immunophenotype-guided interpretable radiomics model for predicting neoadjuvant anti-pd-1 response in stage iii–iv d-mmr/msi-h colorectal cancer. J. Immunother. Cancer.

[80] Seo M.-K., Kang H., Kim S. (2022). Tumor microenvironment-aware, single-transcriptome prediction of microsatellite instability in colorectal cancer using meta-analysis. Sci. Rep..

[81] Saxena S., Jena B., Gupta N., Das S., Sarmah D., Bhattacharya P., Nath T., Paul S., Fouda M.M., Kalra M. (2022). Role of artificial intelligence in radiogenomics for cancers in the era of precision medicine. Cancers.

[82] Bikhchandani M., Amersi F., Hendifar A., Gangi A., Osipov A., Zaghiyan K., Atkins K., Cho M., Aguirre F., Hazelett D. (2023). Pole-mutant colon cancer treated with pd-1 blockade showing clearance of circulating tumor DNA and prolonged disease-free interval. Genes.

[83] Koi M., Okita Y., Carethers J.M. (2018). Fusobacterium nucleatum infection in colorectal cancer: Linking inflammation, DNA mismatch repair and genetic and epigenetic alterations. J. Anus Rectum Colon..

[84] Fessler J., Matson V., Gajewski T.F. (2019). Exploring the emerging role of the microbiome in cancer immunotherapy. J. Immunother. Cancer.

[85] Wong C.C., Yu J. (2023). Gut microbiota in colorectal cancer development and therapy. Nat. Rev. Clin. Oncol..

[86] Borges-Canha M., Portela-Cidade J.P., Dinis-Ribeiro M., Leite-Moreira A.F., Pimentel-Nunes P. (2015). Role of colonic microbiota in colorectal carcinogenesis: A systematic review. Rev. Española De. Enfermedades Dig..

[87] Hu C., Cai D., You W., Zhou Z., Liu J., Li C.-H., Lv M.-Y., Gai B.-W., Chen C., Huang X. (2026). Multi-omics driven immune classification of colorectal cancer: Implications for immunotherapy efficacy prediction and enhancement with wnt signaling inhibition. Cancer Lett..

[88] Zhou Y., Liu J., Shi B., Ma T., Yu P., Li J., Gu Y., Zhang Y. (2025). Evaluation of pan-cancer immune heterogeneity based on DNA methylation. Genes.

[89] Wang Y., Chen P.M., Liu R.B. (2018). Advance in plasma sept9 gene methylation assay for colorectal cancer early detection. World J. Gastrointest. Oncol..

[90] Ratti M., Lampis A., Ghidini M., Salati M., Mirchev M.B., Valeri N., Hahne J.C. (2020). Micrornas (mirnas) and long non-coding rnas (lncrnas) as new tools for cancer therapy: First steps from bench to bedside. Target. Oncol..

[91] Agüera-Sánchez A., Peña-Ros E., Martínez-Martínez I., García-Molina F. (2026). Comprehensive landscape of diagnostic, prognostic and predictive biomarkers in colorectal cancer: From genomics to multi-omics integration in precision medicine. J. Pers. Med..

[92] Bichalski B., Bichalska-Lach M., Waniczek D. (2025). Diagnostic pathways and molecular biomarkers in colorectal cancer: Current evidence and perspectives in poland. Curr. Issues Mol. Biol..

[93] Liu H., Ibrahim E.I.K., Centanni M., Sarr C., Venkatakrishnan K., Friberg L.E. (2025). Integrated modeling of biomarkers, survival and safety in clinical oncology drug development. Adv. Drug Deliv. Rev..

[94] Khazaei Z., Pouliot F., Archambault L. (2026). Biomarkers for precision prognosis in prostate cancer: Imaging, molecular, and integrated approaches. Cancers.

[95] Guo Z., Xu C., Zhang S., Hao Y., Hu X., Zhao M., Xiang C., Piao Y., Sun P., Xiang X. (2026). Expert consensus on the detection and clinical application of tumor mutational burden. Cancer Biol. Med..

[96] Ma X., Dong L., Liu X., Ou K., Yang L. (2022). Pole/pold1 mutation and tumor immunotherapy. J. Exp. Clin. Cancer Res..

[97] Mohiuddin M. (2025). Monitoring and assessment of circulating tumor DNA in cancers using ultrarapid sensitivity as an innovative practice. Health Sci. Rep..

[98] Shi C., Qin K., Lin A., Jiang A., Cheng Q., Liu Z., Zhang J., Luo P. (2022). The role of DNA damage repair (ddr) system in response to immune checkpoint inhibitor (ici) therapy. J. Exp. Clin. Cancer Res..

[99] Qiao D., Wang R.C., Wang Z. (2025). Precision oncology: Current landscape, emerging trends, challenges, and future perspectives. Cells.

[100] Harvey A., Walser E., Lahamm-Andraos R., Yeo C., Wolfe S., Stretch C., Craig S., Bathe O.F. (2026). Leveraging the transcriptome-phenotype relationship to guide clinical management of papillary thyroid cancer. Front. Endocrinol..

[101] Jia Q., Wang A., Yuan Y., Zhu B., Long H. (2022). Heterogeneity of the tumor immune microenvironment and its clinical relevance. Exp. Hematol. Oncol..

[102] Alum E.U. (2025). Ai-driven biomarker discovery: Enhancing precision in cancer diagnosis and prognosis. Discov. Oncol..

[103] Shariaty F., Pavlov V. (2025). Radiogenomics: Transforming lung cancer care through non-invasive imaging and genomic integration. Med. Oncol..

[104] Częścik U., Gryglas M., Szterk A., Flis S. (2026). Evolution or revolution in colorectal cancer treatment: Present and future of new therapeutic options. A Narrat. review. Oncol. Res..

[105] Paduraru D.N., Palcău A.C., Gorecki G.P., Dinulescu A., Băean M.L. (2026). Liquid biopsy in colorectal cancer: Future perspectives through the lens of artificial intelligence-a comprehensive review of novel literature. Int. J. Mol. Sci..

[106] Suehnholz S.P., Nissan M.H., Zhang H., Kundra R., Nandakumar S., Lu C., Carrero S., Dhaneshwar A., Fernandez N., Xu B.W. (2024). Quantifying the expanding landscape of clinical actionability for patients with cancer. Cancer Discov..

[107] Vikas P., Messersmith H., Compton C., Sholl L., Broaddus R.R., Davis A., Estevez-Diz M., Garje R., Konstantinopoulos P.A., Leiser A. (2023). Mismatch repair and microsatellite instability testing for immune checkpoint inhibitor therapy: Asco endorsement of college of american pathologists guideline. J. Clin. Oncol..

[108] Allison K.H., Hammond M.E.H., Dowsett M., McKernin S.E., Carey L.A., Fitzgibbons P.L., Hayes D.F., Lakhani S.R., Chavez-MacGregor M., Perlmutter J. (2020). Estrogen and progesterone receptor testing in breast cancer: Asco/cap guideline update. J. Clin. Oncol..

[109] Leite L.F., Noronha M.M., de Menezes J.S.A., da Conceição L.D., Almeida L.F.C., Cappellaro A.P., Belotto M., de Castria T.B., Peixoto R.D.A., Megid T.B.C. (2025). Anti-egfr therapy in metastatic colorectal cancer: Identifying, tracking, and overcoming resistance. Cancers.

[110] Bhullar D.S., Barriuso J., Mullamitha S., Saunders M.P., O’Dwyer S.T., Aziz O. (2019). Biomarker concordance between primary colorectal cancer and its metastases. EBioMedicine.

[111] Hutomo Y. (2026). Integrasi biomarker dan imaging untuk skrining presisi kanker kolorektal: Tinjauan literatur. J. Ris. Kedokt..

[112] Dasari A., Lin Y., Kopetz S., Jacobs S.A., Lucas P.C., Sahin I.H.H., Deming D.A., Philip P.A., Hong T.S., Wolmark N. (2022). Nrg-gi008: Colon adjuvant chemotherapy based on evaluation of residual disease (circulate-us). Am. Soc. Clin. Oncol..

[113] Vignot S., Lefebvre C., Frampton G.M., Meurice G., Yelensky R., Palmer G., Capron F., Lazar V., Hannoun L., Miller V.A. (2015). Comparative analysis of primary tumour and matched metastases in colorectal cancer patients: Evaluation of concordance between genomic and transcriptional profiles. Eur. J. Cancer.

[114] Tie J., Joshua D.C., Lahouel K., Serigne N.L., Wang Y., Kosmider S., Wong R., Shapiro J., Lee M., Harris S. (2022). Circulating tumor DNA analysis guiding adjuvant therapy in stage ii colon cancer. N. Engl. J. Med..

[115] Chalabi M., Yara L.V., Pedro B.T., Balduzzi S., Van Lent Anja U., Grootscholten C., Dokter S., Nikè V.B., Brechtje A.G., Kuhlmann K. (2024). Neoadjuvant immunotherapy in locally advanced mismatch repair–deficient colon cancer. New Engl. J. Med..

[116] Sun H., Lyu P., Yang S., Wang F., Zhai S., Zhao M., Yuan W., Zhou Q. (2025). Immunotherapy versus chemotherapy as adjuvant therapy for resected msi-h/dmmr colorectal cancer: Real-world evidence informing precision strategies. Front. Immunol..

[117] Pataky R.E., Weymann D., Bosdet I., Yip S., Bryan S., Sadatsafavi M., Peacock S., Regier D.A. (2024). Real-world cost-effectiveness of panel-based genomic testing to inform therapeutic decisions for metastatic colorectal cancer. J. Cancer Policy.

